# Suppressing protein damage response to overcome multidrug resistance in cancer therapy

**DOI:** 10.1038/s41421-025-00826-9

**Published:** 2025-09-30

**Authors:** Fangyuan Shao, Zongjie Li, Hao Xiao, Yujun Chen, Yuheng Zhang, Ling Li, Yuzhong Peng, Xinyi Li, Yuxing Hou, Bo Li, Xiangpeng Chu, Maoxin Ran, Dongyang Tang, Xi Han, Jiaxin Yao, Cuiting Zhang, Lijian Wang, Haifeng Li, Nan Shao, Kai Miao, Xiaoling Xu, Yanxia Shi, Changhua Zhang, Jun Yan, Ying Lin, Chu-Xia Deng

**Affiliations:** 1 Cancer Center, Faculty of Health Sciences, University of Macau, Macau SAR, China; 2 Center for Precision Medicine Research and Training, Faculty of Health Sciences, University of Macau, Macau SAR, China; 3 MoE Frontiers Science Center for Precision Oncogene, University of Macau, Macau SAR, China; 4 Guangdong Key Laboratory of Animal Breeding and Nutrition, Institute of Animal Science, Guangdong Academy of Agricultural Sciences, Guangzhou, Guangdong China; 5 Guangdong-Hong Kong-Macau University Joint Laboratory of Digestive Cancer Research, Guangdong Provincial Key Laboratory of Digestive Cancer Research, Scientific Research Center, The Seventh Affiliated Hospital of Sun Yat-sen University, Shenzhen, Guangdong China; 6 Department of Colorectal Surgery, Fujian Medical University Union Hospital, Fuzhou, Fujian China; 7 Department of Medical Oncology, Sun Yet-sen University Cancer Center, Collaborative Innovation Center of Cancer Medicine, State Key Laboratory of Oncology in South China, Sun Yat-sen University, Guangzhou, Guangdong China; 8 Department of Breast Surgery, The First Affiliated Hospital, Sun Yat-sen University, Guangzhou, Guangdong China; 9 Zhuhai UM Science & Technology Research Institute, Hengqin, Zhuhai, Guangdong China

**Keywords:** Cancer therapeutic resistance, Proteasome

## Abstract

Multidrug resistance is a significant barrier in cancer therapy largely due to poorly understood regulatory mechanisms. Here we reveal that certain anticancer drugs can bind to newly synthesized proteins prior to reaching their canonical targets, resulting in various forms of protein damage. This binding disrupts protein functions, particularly those of mitochondrial proteins, resulting in substantial cytotoxicity. The protein damage is further exacerbated by mitochondrial reactive oxygen species generated as a consequence of the initial damage, creating a positive feedback loop. In response, cancer cells rapidly initiate a chain of events, which we term the Protein Damage Response (PDR). This includes damage recognition primarily mediated by protein ubiquitination and subsequent damage clearance via the proteasome system. Notably, patients with advanced, drug-resistant metastatic breast or colon cancers exhibit elevated proteasome activity. In an effort to predict drug resistance, we developed a sensitive kit for detecting proteasome levels, enabling the identification and subtyping of patients with high proteasome activity to support tailored therapeutic strategies. Using a three-dimensional tumor slice culture-based drug sensitivity assay and an investigator-initiated clinical trial, we demonstrate that three clinically approved proteasome inhibitors effectively overcome multidrug resistance in colon and breast cancer patients with elevated proteasome activity.

## Introduction

According to the estimates by the International Agency for Research on Cancer, there were 19.96 million new cancer cases and 9.74 million cancer deaths worldwide in 2022^1^. One of the main reasons for the high cancer mortality rate is that many patients develop resistance to drugs during drug treatment^2,3^, which leads to drug treatment failure or reduced curative effects and aggravates the development of tumors^4–6^.

Some patients exhibit innate drug resistance; that is, these patients have no response to anticancer drugs at the beginning of treatment. In many other cases, drug resistance is acquired during the treatment process, i.e., patients show a good therapeutic effect in the beginning but gradually lose responsiveness during chemotherapy^4,7–9^. Even more difficult to treat, patients who acquire tolerance to therapeutic drugs often develop cross-resistance to other drugs that they have never been exposed to, even those with different structures and mechanisms of action — a phenomenon called multidrug resistance (MDR)^2,3,5^.

Recent studies have shown that the mechanisms of MDR^6^ involve DNA damage repair, drug target mutation, drug metabolism and inactivation, drug efflux, aberrant cell death regulation, cancer stem cells, etc.^5^. To identify specific targets and pathways that may overcome drug resistance, we previously utilized RNAi-mediated gene knockdown and high-throughput drug library screening in multiple cancer cell lines to identify top candidates that were then subjected to in vivo validation^7,10,11^. In a cisplatin (CIS)-resistant cell line obtained by an increasing CIS concentration gradient, we found that it was also cross-resistant to > 40 anticancer drugs tested. Notably, the MDR of these cells, which was accompanied by high levels of proteasome activity, could be overcome by blocking proteasome activity with a small-molecule inhibitor, bortezomib. Because a main function of proteasome is to destroy damaged proteins, we hypothesized that protein damage might serve as a common cytotoxic mechanism by which anticancer drugs kill cancer cells. To investigate this further, we monitored 11 structurally unrelated drugs and found six of them resulted in significantly increased protein ubiquitination^7^. Although these data are consistent with a view that anticancer drugs might cause protein damage, it does not exclude some other alternative explanations, as protein ubiquitination also plays a role in regulating protein activity and localization in addition to mediating protein degradation^12,13^. Thus, this early study, while reveals an intriguing issue, also raises many crucial questions that are largely unexplored/unanswered. Are the ubiquitinated proteins indeed damaged proteins caused by anticancer drugs? If so, what are the types of damages and how they occur? If protein damaging is a new mechanism of action of anticancer drugs, how is it related to the canonical targets of these drugs? What are the cellular responses that can effectively compensate for the cytotoxicity associated with protein damaging, eventually leading to MDR? Importantly, how to use the new knowledge to predict and overcome MDR?

To address these questions, we employed a large-scale screening using 101 anticancer drugs for various forms of protein damages and found that the majority of the agents could cause protein structural changes, protein misfolding, and oxidative damage. Mechanistically, neosynthesized proteins are the primary source of these damaged proteins, resulting from the large-scale binding of anticancer drugs to them, which leads to profound cytotoxicity. To counteract this effect, cells quickly initiate a cellular response, which we define as the Protein Damage Response (PDR). This response primarily involves damage recognition through protein ubiquitination and damage clearance via the proteasome, which protects cells from death and contributes to resistance against anticancer drugs.

## Results

### The vast majority of anticancer drugs induce a wide range of protein damage

**Fig. 1:**
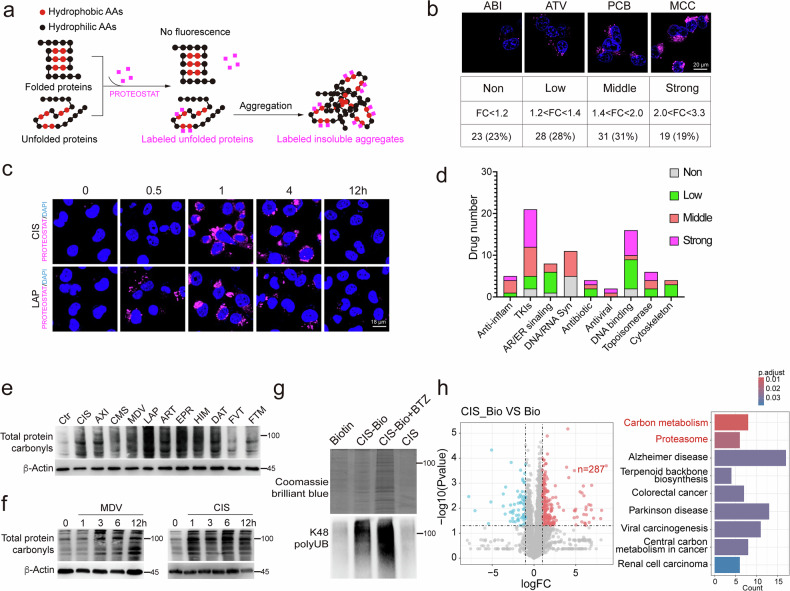

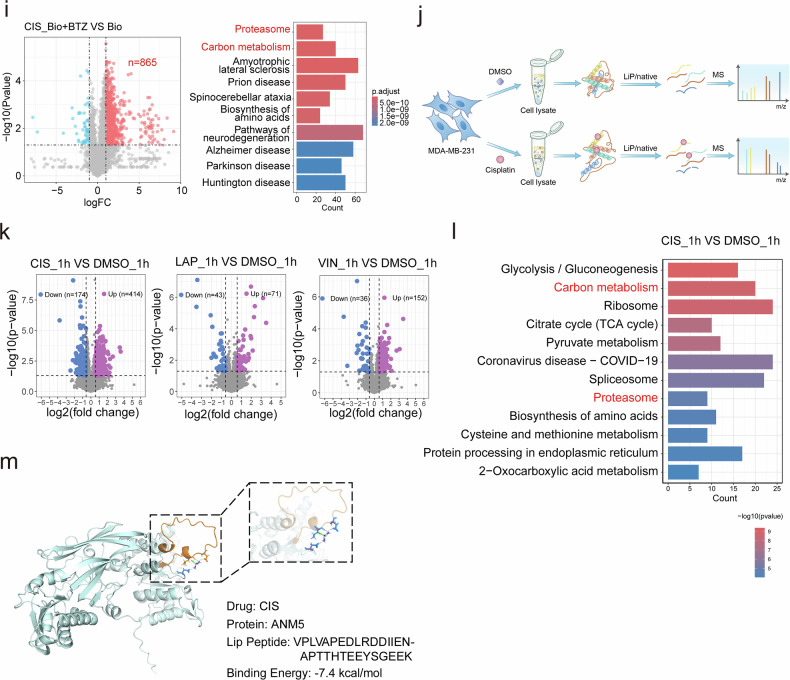
The vast majority of anticancer drugs induce a wide range of protein damage. **a** Schematic diagram of PROTEOSTAT staining for unfolded and aggregated proteins. **b** MDA-MB-231 cells were treated with 101 anticancer drugs for 1 h, and protein aggregation was tested by the PROTEOSTAT staining. Drugs were classified into subtypes based on *P* values which ranged from 0.63 to 1.86e–06. **c** MDA-MB-231 cells were treated with CIS or LAP for indicated time, and protein aggregation was tested by the PROTEOSTAT staining. **d** The working mechanisms of 101 anticancer drugs and the associated types of protein damage. **e** MDA-MB-231 cells were treated with indicated anticancer drugs (*n* = 11) for 8 h, and the total protein carbonylation was detected by western blotting. **f** MDA-MB-231 cells were treated with MDV or CIS for indicated time, and total protein carbonylation was detected by western blotting. **g**, **h** MDA-MB-231 cells were treated with indicated drugs for 3 h, and biotin was pulled down. Interacting proteins were stained with Coomassie brilliant blue and K48 polyUB was detected by western blotting (**g**). CIS-biotin and biotin-pulled down proteins were analyzed by MS. Enhanced binding proteins (red) were highlight by log_2_FC > 1, *P* value < 0.05 and analyzed by KEGG enrichment (**h**). **i** CIS-biotin treatment was further combined with BTZ, and CIS-biotin-pulled down proteins were analyzed by MS. Enhanced binding proteins (red) were highlight by log_2_FC > 1, *P* value < 0.05 and analyzed by KEGG enrichment. **j** Flow chart depicting the LiP-MS assay. MDA-MB-231 cells were treated with CIS or DMSO for 1 h, followed by treatment with proteinase K (PK), LysC, and trypsin digestion and analysis by MS. Structure changes caused by CIS treatment and the binding of CIS either increase or prevent PK digestion, respectively, leading to differential MS peptide profiles. **k**, **l** MDA-MB-231 cells were treated with CIS, LAP, or VIN for 1 h, and proteins with differential MS peptide profiles were identified (**k**) and analyzed by KEGG enrichment (**l**). **m** PyMOL analysis of protein steric structure showing peptides identified by Lip-MS. Molecular docking predicted a CIS-binding energy of –7.4 kcal/mol.

To investigate whether anticancer drugs could damage proteins, we conducted a large-scale screening with 101 FDA-approved anticancer drugs in MDA-MB-231 cells for the detection of protein damage (Supplementary Table S1). The MDA-MB-231 cells were treated with each drug at its IC_50_ concentration for 1 h, and protein damage was first evaluated based on protein misfolding. During protein misfolding, hydrophobic amino acids that are normally hidden in the core of folded proteins become disordered and exposed. This exposure leads to fluctuations in local polarity, which can be detected by molecular rotor dyes like PROTEOSTAT (Fig. 1a)^14,15^. Compared to the untreated controls, we found 87% of anticancer drugs caused protein aggregation at 1 h post treatment, with 19%, 31% and 28% of anticancer drugs causing strong, middle, and low levels of protein aggregation, respectively (Fig. 1b and Supplementary Fig. S1a). Then we tested the dynamics of protein aggregation induced by drugs, and the data indicated that Lapatinib (LAP), a small-molecule tyrosine kinase inhibitor of EGFR and HER2, caused protein aggregation at 30 min and that CIS caused protein aggregation at 1 h. Protein aggregations caused by both drugs were cleared away at 12 h (Fig. 1c). Then we analyzed the anticancer drugs targeting nine biological processes and found that they all could damage proteins at varying degrees (Fig. 1d and Supplementary Fig. S1b).

Next, we investigated whether the anticancer drugs could cause oxidative damage by examining protein carbonyls, which are formed from ketones (>C=O) and aldehydes (−CH=O) arising from the amine, guanidino, or hydroxyl groups in amino acid side chains. The data indicated that all 34 drugs tested induced varying degrees of oxidative damage 8 h after the treatment (Fig. 1e and Supplementary Fig. S1c). A time course study indicated that MDV3100 (MDV), an androgen receptor antagonist, induced protein carbonyls in a time-dependent manner starting from 3 h post treatment, and that CIS-induced protein carbonyls reached the maximum at 1 h post treatment and maintained saturated at later time points (Fig. 1f).

Since the majority of anticancer drugs damaged proteins, we suspected that they might be able to bind proteins. To investigate this, we tested the CIS, which is a platinum compound covalently binding to guanine and adenine residues of DNA^16^. We linked CIS to biotin to create CIS-biotin and treated MDA-MB-231 cells for 3 h to bind proteins. Afterward, we used streptavidin immobilized on agarose to pull down CIS-biotin, and the bound proteins were visualized using Coomassie brilliant blue staining (Fig. 1g). The results showed that CIS-biotin pulled down more proteins than biotin alone. The proteins pulled down by CIS-biotin may be damaged, as indicated by increased ubiquitination. Mass spectrometry analysis identified 287 proteins (log_2_FC > 1, *P* value < 0.05) pulled down by the CIS-biotin after extracting proteins bound by biotin alone (Fig. 1h). Because we believed that CIS could bind to and damage proteins, we next treated the CIS-biotin-treated cells with bortezomib (BTZ) to block the degradation of the damaged proteins. The data showed that the number of proteins increased markedly, reaching up to 865 (Fig. 1i), confirming the notion that CIS binds to and damages proteins. Notably, the proteins involved in “carbon metabolism”, many of which are associated with mitochondrial activity, as well as those related to the term “proteasome”, which is responsible for the clearance of damaged proteins, were enriched among the proteins bound by CIS (Supplementary Table S2). Consistent with these findings, biotin alone did not cause protein aggregation. In contrast, CIS-biotin induced protein aggregation at 3 h, with partial recovery observed at 6 h (Supplementary Fig. S1d). Because the large quantity of proteins is involved with a short time window, we believed that the drug might bind to proteins nonspecifically.

The binding of anticancer drugs to proteins might change the local amino acid polarity and interfere with the protein folding and structure. To investigate this, we conducted limited proteolysis-coupled mass spectrometry (LiP-MS) after CIS treatment to identify the CIS-binding sites and protein structure changes at the proteome-wide scale^17^. In this workflow, MDA-MB-231 cells were treated with CIS or vehicle (DMSO) for 1 h, followed by limited proteolysis with proteinase K, which generated protein fragmentation patterns dependent on the structural features of proteins and/or their drug-binding sites. The resulting structure-specific protein fragments were then processed through a standard proteomics sample preparation workflow to produce peptides amenable to liquid chromatography-tandem mass spectrometry (LC-MS/MS) analysis (Fig. 1j). By applying a log_2_FC threshold of ± 0.5 and an adjusted *P* value threshold of 0.05, we identified 414 proteins exhibiting enhanced proteolysis and 174 proteins exhibiting reduced proteolysis, indicating structural alterations and drug-binding site modifications, respectively (Fig. 1k). Further bioinformatics analysis revealed predominant enrichment in biological processes related to mitochondrial function and the proteasome (Fig. 1l), which is consistent with the results of the CIS-biotin pull down assay (Fig. 1h and Supplementary Table S2). We further tested LAP, vincristine (VIN), epirubicin (EPR), and gemcitabine (GEM) treatment, identifying 114, 188, 113, and 166 proteins with structural changes, respectively (Fig. 1k and Supplementary Fig. S1e, g). Peptides bound by CIS were visualized using PyMOL in the protein’s steric structure, revealing that CIS has moderate binding affinity to the Lip-MS-identified peptide (–7.4 kcal/mol, Fig. 1m). Similar results were observed within 1 h of LAP or VIN treatment (Supplementary Fig. S1f). Altogether, our study from multiple angles, including protein misfolding, aggregation, oxidative damage and structural change, demonstrated that a majority (77% of 101) of the anticancer drugs tested damage the protein, which is a phenomenon rarely recognized but commonly occurs upon treatment with these drugs.

### Anticancer drugs damage neosynthesized proteins and their functions

**Fig. 2:**
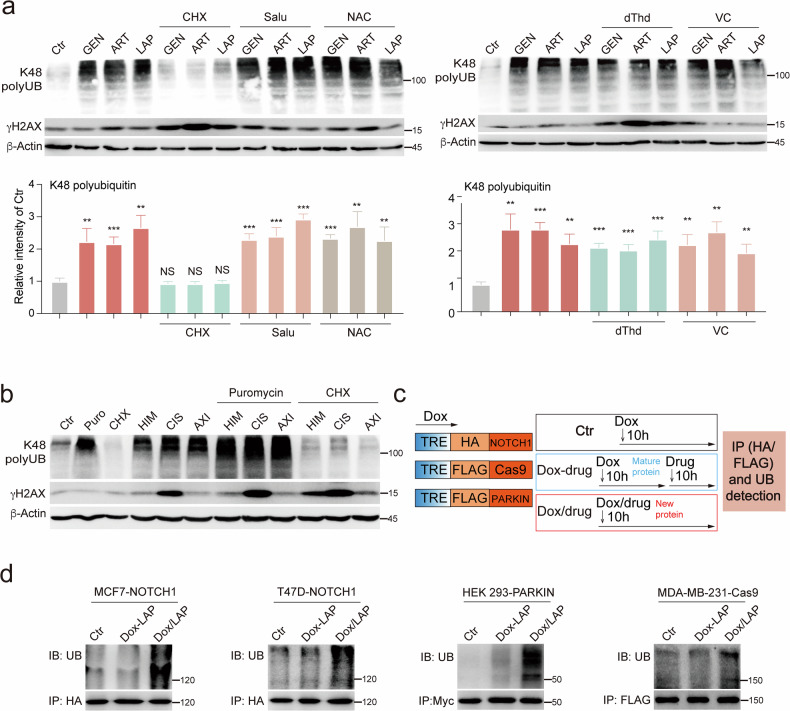

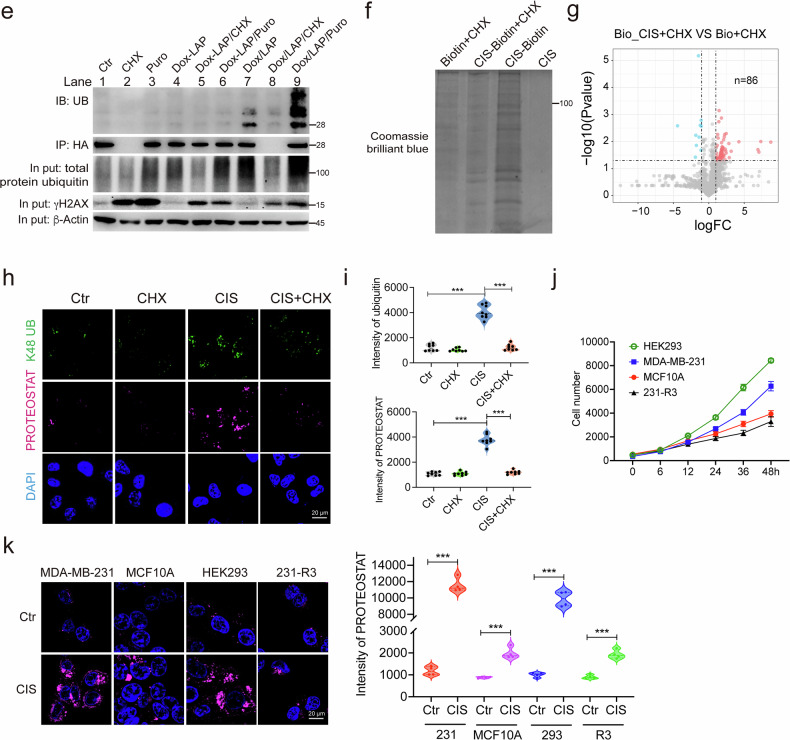
Anticancer drugs damage neosynthesized proteins and compromise their functions. **a** MDA-MB-231 cells were treated with indicated anticancer drugs alone or in combination with various inhibitors, respectively. K48 polyUB and γH_2_AX were detected by western blotting, and quantification for ubiquitin was shown below. **b** MDA-MB-231 cells were treated with indicated anticancer drugs alone or in combination with CHX or Puro for 1 h, and K48 polyUB and γH_2_AX were detected by western blotting. **c** A treatment model was used to compare the damage between matured proteins and neosynthesized proteins. **d** MCF7, T47D, HEK293, and MDA-MB-231 cells transfected with Dox-inducible Notch1, Parkin, or Cas9, respectively. Cells were treated with LAP as indicated, and Notch1, Parkin, or Cas9 were pulled down for ubiquitination analysis. **e** MDA-MB-231-GFP cells were treated with LAP alone or in combination with protein synthesis inhibitors for 10 h, and GFP was pulled down for detection of K48 polyUB. Input proteins were tested for K48 polyUB and γH_2_AX. **f**, **g** MDA-MB-231 cells were treated with indicated drugs for 3 h, and CIS-biotin and biotin-pulled down proteins were stained with Coomassie brilliant blue (**f**) and analyzed by MS. Enhanced binding proteins (red) were highlight by log_2_FC > 1, *P* value < 0.05 (**g**). **h**, **i** MDA-MB-231 cells were treated with CIS alone or in combination with CHX, and protein ubiquitination and aggregation were detected by immunofluorescence and PROTEOSTAT staining, respectively (**h**). The intensity of ubiquitin and PROTEOSTAT was quantified (**i**). **j** Proliferation rate of MCF10A, HEK293, MDA-MB-231, and 231-R3 cells was detected by alamar blue assay. **k** MCF10A, HEK293, MDA-MB-231, and 231-R3 cells were treated with CIS for 1 h and protein aggregation was detected by PROTEOSTAT staining. Data are presented as mean value (at least three replicates) ± SD, and *P* value was calculated by comparison with the Ctr group or indicated separately (two-tailed Student’s *t*-test, *P < 0.05, **P < 0.01, ***P < 0.001, NS, no significance).

Next, to understand the mechanism of protein damage induced by anticancer drugs, we tested various inhibitors in combination with anticancer drugs to assess protein damage. Since the levels of CIS-biotin-bound proteins are associated with the accumulation of K48 polyubiquitination (K48 polyUB), we used K48 polyUB as a marker for various forms of protein damage. We found that cycloheximide (CHX), which blocks protein synthesis on the ribosome^18^, completely blocked K48 polyUB induced by 3 anticancer drugs. These drugs were genistein (GEN), artemether (ART), and LAP. Other factors tested showed no obvious effects. These include salubrinal (Salu) that inhibits endoplasmic reticulum stress, thymidine (dThd) that blocks cell cycle progression^19^, as well as *N*-acetyl-l-cysteine (NAC) and l-ascorbic acid (VC) that block reactive oxygen species (ROS) accumulation (Fig. 2a). These inhibitors were also tested on 9 additional drugs that induce K48 polyUB (Supplementary Fig. S2a, b). These data suggest that protein synthesis is the main target of anticancer drug-induced protein damage, and the data also revealed that the anticancer drugs have distinct effects, causing protein damage and DNA damage. For example, CHX blocked protein damage and induced DNA damage, while dThd-induced DNA damage but did not induce protein damage (Fig. 2a).

To further understand the role of protein synthesis in drug-induced protein damage, we compared the effects of CHX and puromycin (Puro). Puro interferes with protein synthesis by incorporating into the C-terminus of nascent polypeptides, leading to premature termination of new peptides^20^. Strikingly, unlike CHX treatment, which completely blocked protein damage induced by all drugs, Puro treatment significantly exacerbated the protein damage caused by the drugs (Fig. 2b). Because Puro treatment generated more prematurely terminated nascent peptides, this may account for the significant increase in protein damage. We further tested Rocaglamide and Anisomycin, which are inhibitors of protein synthesis. Rocaglamide and Anisomycin treatment blocked the protein damage induced by all 9 drugs (Supplementary Fig. S2c). Altogether, our data suggest that newly synthesized proteins might be the main targets of anticancer drug-induced damage. To further investigate this hypothesis, we employed the doxycycline (Dox)-inducible Tet-on system to investigate protein damage during and after the synthesis of several well-known proteins, following the protocol outlined in Fig. 2c. In the first group (Ctr), the tested genes were induced by Dox for 10 h without drug treatment. In the second group (Dox-drug), Dox was used to induce protein synthesis for 10 h, then washed away before LAP treatment for another 10 h. In the third group (Dox/drug), Dox and LAP were added simultaneously to expose newly synthesized proteins to LAP. The tested proteins were then pulled down by immunoprecipitation, and their ubiquitination was detected (Fig. 2c). For all three proteins (HA-NOTCH1, Myc-PARKIN and FLAG-Cas9) tested in 4 cell lines (MCF7, T47D, HEK293, and MDA-MB-231 cells), the results consistently demonstrated that protein damage occurred in the third group (Fig. 2d), i.e., the anticancer drugs preferably damaged newly synthesized proteins but had little effect on the same proteins that were matured. To further demonstrate this, we interfered with the synthesis of GFP by CHX or Puro (Fig. 2e). The LAP treatment-induced ubiquitination of GFP (lane 7) was completely blocked by the inhibition of GFP synthesis (CHX, lane 8) and further increased by the premature termination of GFP synthesis (Puro, lane 9). Comparison of Dox-LAP/Puro (lane 6) and Dox/LAP/Puro (lane 9) treatment clearly demonstrated that LAP/Puro treatment caused a strong increase in total protein damage, while the damage to GFP occurred only in synthesizing proteins (lane 9) and not in already synthesized or matured GFP proteins (lane 6). The damage to NOTCH1, PARKIN, Cas9, and GFP by LAP treatment indicates that anticancer drugs can nonspecifically damage proteins, primarily the nascent peptides and newly synthesized proteins. Because CIS binds to a large number of proteins, potentially including newly synthesized ones, we tested the effect of CHX treatment on CIS-binding proteins. Indeed, the abundance of CIS-biotin-binding proteins was significantly reduced by CHX treatment (Fig. 2f), and binding proteins were decreased from 287 to 86 after the blockage of new protein synthesis (Figs. 1h, 2g). Consistently, the protein aggregation and K48 polyUB induced by CIS treatment were completely blocked by CHX treatment (Fig. 2h, i), further indicating that the anticancer drugs bind to newly synthesized proteins and nascent peptides, leading to protein damage. Given that drug-induced protein damage is primarily associated with de novo protein synthesis, which is heightened in rapidly proliferating cells, we investigated the drug response in non-malignant, drug-sensitive, and drug-resistant cancer cells. CIS treatment induced significantly higher protein aggregation in rapidly proliferating HEK293 and MDA-MB-231 cells, compared to the slower-growing MCF10A and 231-R3 cells (Fig. 2j, k). These results indicate that drug-induced protein damage is associated with cell proliferation, a key characteristic of cancer.

Next, we asked whether the protein damage induced by anticancer drugs could affect protein function. Since ubiquitination levels of the newly synthesized proteins were higher, we tested the function of these proteins by investigating mitochondria-targeted GFP (MTS-GFP) fluorescence intensity and pyruvate dehydrogenase (PDH) activity. MDA-MB-231-MTS-GFP cells were treated with LAP or CIS during MTS-GFP synthesis (Dox/drug) or after MTS-GFP synthesis (Dox-drug). GFP fluorescence was dramatically decreased in the Dox/drug group compared to the Dox-drug group (Supplementary Fig. S2d). There was no significant change in the GFP level in these groups (Supplementary Fig. S2e), suggesting that the decline in GFP fluorescence was caused by protein damage and not by changes in protein abundance. Next, we examined PDH abundance and activity following CHX treatment (Supplementary Fig. S2f). We then conducted the CHX chase assay to monitor the recovery of PDH activity with or without drug treatment. Our results indicate that while PDH protein abundance recovers after CHX washout, CIS or LAP treatment significantly reduced the recovery of PDH enzyme activity (Supplementary Fig. S2g). This provides further evidence that these drugs impair protein function. Altogether, our data demonstrate that anticancer drugs interact nonspecifically with newly synthesized proteins and impair their function rather than affecting their synthesis.

### Anticancer drugs damage neosynthesized proteins prior to the generation of mitochondrial ROS, which in turn enhances protein damage

**Fig. 3:**
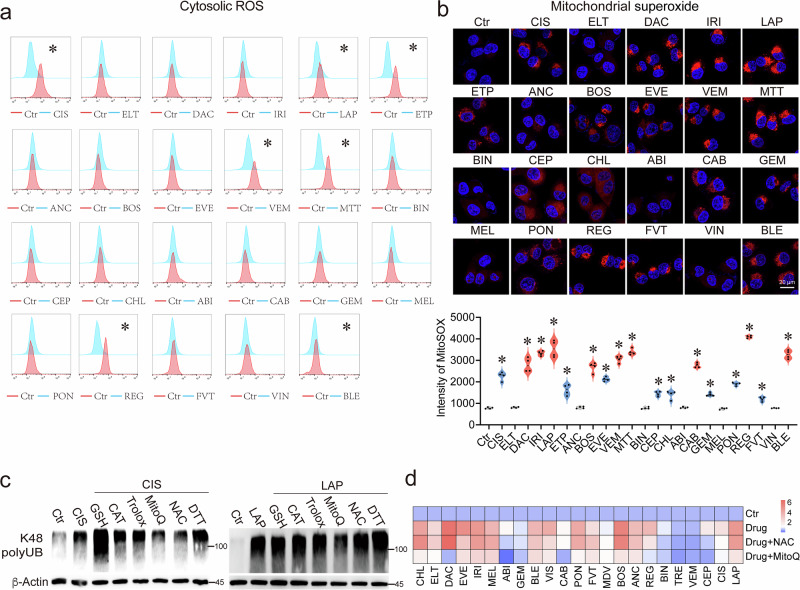

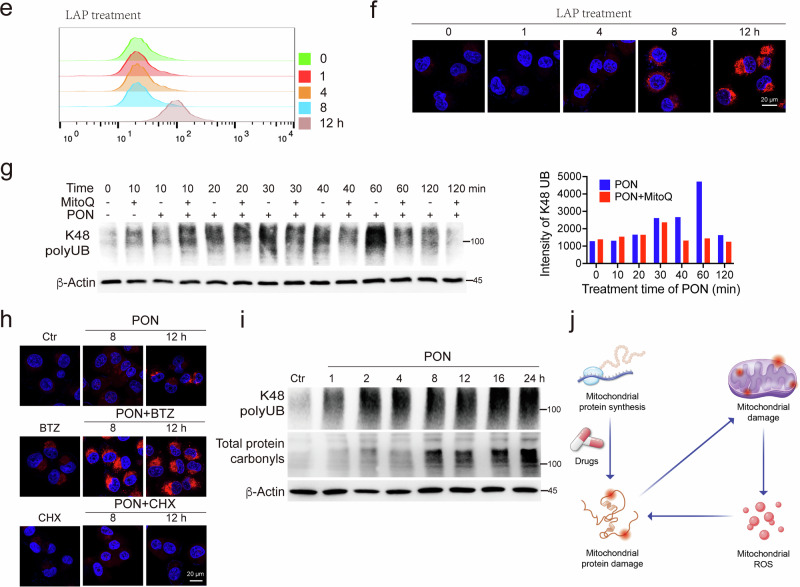
Anticancer drugs damage neosynthesized proteins followed by the generation of mtROS, which in turn enhance protein damage. **a**, **b** MDA-MB-231 cells were treated with indicated anticancer drugs (*n* = 23); cytosol ROS accumulation was detected by DCFDA staining (**a**), and mitochondrial superoxide were detected by MitoSOX™ Red reagent (**b**). **c** MDA-MB-231 cells were treated with indicated drugs alone or in combination with GSH, DTT, Trolox, NAC, ROS catalase, or MitoQ, and K48 polyUB was detected by western blotting. **d** Heatmap showing the intensity of K48 polyUB induced by single drug (*n* = 23) treatment or in combination with NAC or MitoQ treatment. **e**, **f** MDA-MB-231 cells were treated with LAP for indicated time periods, and cytosol ROS accumulation was detected by DCFDA staining (**e**). Mitochondrial superoxide was detected by MitoSOX™ Red reagent (**f**). **g** MDA-MB-231 cells were treated with PON alone or in combination with MitoQ for indicated time periods, and K48 polyUB was detected by western blotting and quantified. **h** MDA-MB-231 cells were treated with PON alone or in combination with BTZ or CHX, and mitochondrial superoxide was detected by MitoSOX™ Red reagent. **i** MDA-MB-231 cells were treated with PON for indicated time periods, and K48 polyUB and protein total carbonylation were detected by western blotting. **j** A summary of the interplay between anticancer drugs and mitochondrial ROS in protein damage. All values are presented as mean value (at least three replicates) ± SD, and *P* value was calculated by comparison with the Ctr group or indicated separately (two-tailed Student’s *t*-test, *P < 0.05).

ROS are believed to directly oxidize biological macromolecules, such as proteins, leading to their damage^21^. To investigate whether ROS play a role in drug-induced protein damage, we first examined the levels of ROS upon the treatment with 23 anticancer drugs at 12 h, and we found that 7 drugs induced cytosolic ROS while 17 drugs induced mitochondrial ROS (mtROS) (Fig. 3a, b). Next, we investigated whether blocking ROS generation affects protein damage dynamics by comparing the effects of inhibitors on cytosolic and mtROS. Notably, we found that MitoQ, which is an mtROS inhibitor, was the most potent in blocking drug-induced protein damage compared with the other 5 cytosolic ROS inhibitors (Fig. 3c) and could attenuate the protein damage induced by 20 drugs tested, while NAC could only mildly attenuate protein damage for 8 drugs (Fig. 3d and Supplementary Fig. S3a, b). These data indicated that mtROS are more commonly generated and contribute to drug-induced protein damage.

Since our earlier finding indicates that anticancer drugs directly damage the neosynthesized proteins at early phase, we tested the time of ROS generation. We found that cytosolic ROS and mtROS were observed at 8–12 h post treatment (Fig. 3e, f and Supplementary Fig. S3c). In contrast, 21 out of 23 drugs caused protein damage within 1 h (Supplementary Fig. S1a), indicating that anticancer drugs induced protein damage earlier than the generation of ROS, which contributed to damage at a later stage. To validate this, we tested ponatinib (PON) and found that PON-induced protein damage started at 10 min, while the effect of MitoQ on protein damage was not observed until 40 min later and became pronounced at 60 min (Fig. 3g). We then compared MitoQ and CHX treatments using 8 drugs. The results indicated that MitoQ only partially blocked the protein damage, whereas CHX treatment completely prevented it (Supplementary Fig. S3b). Collectively, these results suggested that mtROS caused protein damage at a later stage than the initial damage induced by the drugs on neosynthesized proteins.

Next, we studied the relationship between anticancer drug-induced damage on neosynthesized proteins and mtROS. Since 99% of mitochondrial proteins are synthesized in cytosolic ribosomes and imported into the mitochondria in a linear manner^22^, newly synthesized mitochondrial proteins are particularly vulnerable to drug treatment. Indeed, our CIS-biotin-binding proteome, Lip-MS, and ubiquitin-proteome analyses identified 34, 67, and 71 mitochondrial proteins, respectively (Supplementary Table S2). These mitochondrial proteins were damaged or labeled with ubiquitin, including respiratory chain complexes I to III, which are the major sources of mtROS generation^23,24^. To validate the causal relationship between the damage on neosynthesized proteins and mtROS, we tested several drugs, including PON. We found that increased protein damage by BTZ combination treatment significantly enhanced PON-induced mtROS. In contrast, blocking the protein damage with CHX treatment eliminated PON-induced mtROS (Fig. 3h). These results indicate that anticancer drugs first induce damage to newly synthesized proteins, which in turn causes the generation of mtROS. Notably, we observed that PON treatment induced protein ubiquitination at 1 h, while protein carbonylation was detected at 8 h (Fig. 3i). Collectively, these data suggest a positive feedback loop between protein damage and mtROS: anticancer drugs damage newly synthesized proteins, particularly mitochondrial proteins, in the early phase of treatment, leading to mtROS generation that subsequently exacerbates the protein damage (Fig. 3j).

### Anticancer drug treatment triggers a ubiquitin-proteasome-dependent PDR

**Fig. 4:**
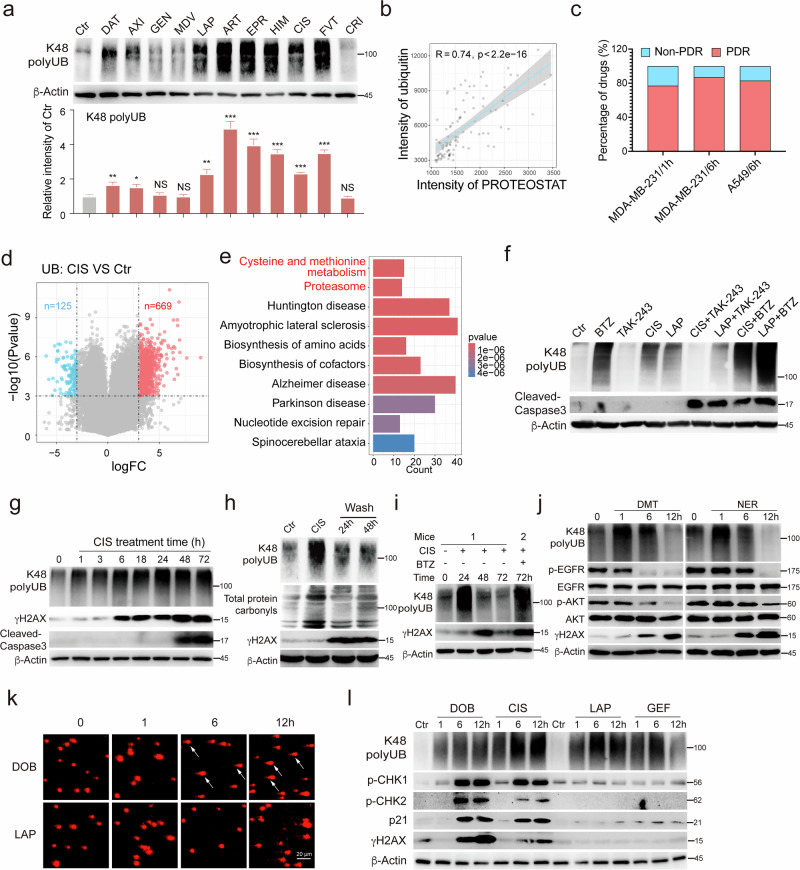
Anticancer drug treatment triggers the ubiquitination and proteasome-dependent PDR. **a** MDA-MB-231 cells were treated with indicated anticancer drugs (*n* = 11) for 1 h, and K48 polyUB was detected by western blotting. Quantifications of different damages were shown below. **b** Correlation between 101 anticancer drugs-induced protein aggregation and K48 polyUB. **c** Statistics of drugs-induced K48 polyUB in MDA-MB-231 cells at 1 h (*n* = 101) and 6 h (*n* = 115), and that in A549-6 h (*n* = 123). **d**, **e** Volcano plot showing changes in ubiquitinated protein levels after 6 h of CIS treatment (**d**), and the proteins with upregulated ubiquitination were analyzed using KEGG enrichment (**e**). **f** MDA-MB-231 cells were treated with indicated anticancer drugs alone or in combination with BTZ or TAK-243, and K48 polyUB and cleaved caspase 3 were detected by western blotting. **g** MDA-MB-231 cells were treated with CIS for indicated time periods, and K48 polyUB, γH_2_AX, and cleaved caspase 3 were detected by western blotting. **h** MDA-MB-231 cells were treated with CIS for 6 h and washed, and K48 polyUB, protein carbonylation, and γH_2_AX were detected by western blotting at 24 h and 48 h. **i** Nude mice bearing MDA-MB-231 xenograft tumors were treated with vehicle, CIS (3 mg/kg) or BTZ (1 mg/kg), and K48 polyUB and γH_2_AX were detected for tumors at 24 h, 48 h, and 72 h. Tumor tissues were harvested by needle biopsy from mouse 1 at 0, 24 and 48 h. Both mice 1 and 2 were sacrificed at 72 h for tumor collection. **j** MDA-MB-231 cells were treated with 2 EGFR inhibitors for indicated time periods, and K48 polyUB, p-EGFR, EGFR, p-AKT, AKT, and γH_2_AX were detected by western blotting. **k** MDA-MB-231 cells were treated with DOB or LAP, and DNA damage was detected by comet assay. **l** MDA-MB-231 cells were treated with indicated drugs, and K48 polyUB, p-CHK1, p-CHK2, p21, γH_2_AX were detected by western blotting. All values are presented as mean value (at least three replicates) ± SD, and *P* value was calculated by comparison with the Ctr group or indicated separately (two-tailed Student’s *t*-test, *P < 0.05, **P < 0.01, ***P < 0.001, NS, no significance).

Our earlier data indicated that the inhibition of proteasome activity by BTZ led to further accumulation of K48 polyUB (Fig. 1g) and an increased number of proteins bound by CIS (Fig. 1h, i). This suggests that the host cells might have initiated a response mediated by the ubiquitin-proteasome system to clear away damaged proteins, which we termed the PDR. To further investigate the causal relationship between anticancer drugs-induced protein damage and the PDR, we conducted a screening with the 101 drugs in MDA-MB-231 cells for detection of K48 polyUB. We found that the majority of the anticancer drugs induced K48 polyUB in 1 h (Fig. 4a and Supplementary Fig. S4a), which is positively correlated with the drugs-induced protein damage (Fig. 4b). Six hours of drug treatment further increased the K48 polyUB rate from 77% to 87% in MDA-MB-231 cells (Fig. 4c and Supplementary Fig. S4a, b). We also observed that 83% of the drugs induced K48 polyUB in A549 cells at 6 h post treatment (Fig. 4c and Supplementary Fig. S4c). To study this in detail, we conducted an analysis of the ubiquitin-modified proteome following CIS treatment. Our assay demonstrated high reproducibility between biological replicates, identifying ubiquitination sites for 59,340 peptides corresponding to 7767 distinct proteins upon CIS treatment, compared to 47,376 peptides corresponding to 6988 distinct proteins without treatment. Following CIS treatment, we identified 1102 peptides corresponding to 669 proteins (log_2_FC > 3, *P* value < 0.05) that exhibited increased ubiquitin conjugation compared to the vehicle treatment (Fig. 4d). Notably, consistent with the CIS-biotin-binding proteome and the LiP-MS structural proteome, terms related to “cysteine and methionine metabolism,” primarily from the mitochondria, as well as “proteasome”, were highly enriched among the 669 ubiquitinated proteins (Fig. 4e and Supplementary Table S2). These data indicate that ubiquitination serves as an early step in marking or recognizing damaged proteins. To confirm this, we treated cells with TAK-243, an inhibitor of E1 enzyme activity^25^, and found that CIS- and LAP-induced K48 polyubiquitination was completely blocked (Fig. 4f). Next, we inhibited damaged protein clearance via BTZ treatment, leading to a significant accumulation of CIS- and LAP-induced K48 polyUB. Notably, blocking either process resulted in a dramatic activation of caspase 3 (Fig. 4f). To mitigate potential off-target effects of proteasome inhibitors, we further investigated the knockdown or knockout of PSMB7 and PSMB5, respectively. Suppression of either PSMB7 or PSMB5 resulted in enhanced K48 polyUB and protein aggregation caused by drug treatment (Supplementary Fig. S4d, e).

Many anticancer drugs have their own primary targets. For example, CIS kills cancer cells by damaging DNA through inducing DNA-adducts^26^. Since our data revealed that protein damage induced by virtually all anticancer drugs occurs quickly upon treatment, the relationship between protein damage and the well-known targets of these drugs remains unknown. To investigate this, we first compared dynamic progressions of protein damage and DNA damage induced by CIS and the data revealed a time-dependent increase in protein damage starting at 1 h, and DNA damage appeared several hours later (Fig. 4g). To study the prolonged effects of CIS, we washed it away after 6 h of treatment. We observed increased protein damage at this time point, which was subsequently decreased (Fig. 4h). In contrast, strong DNA damage was observed at 24 h and persisted for 48 h, reflecting the distinct mechanisms by which cells manage protein damage and DNA damage. In the xenograft tumors, CIS induced strong protein damage at 24 h, and the damaged proteins were gradually cleared away from 24 h to 72 h, while DNA damage was observed at 48 h and repaired at 72 h (Fig. 4i). Meanwhile, BTZ treatment caused the accumulation of both protein and DNA damage (Fig. 4i). These results indicate that CIS may target the proteins to induce damage prior to affecting their canonical targets. To further demonstrate this, we tested the neratinib (NER) and dacomitinib (DMT), which are selective inhibitors for the epidermal growth factor receptor (EGFR)^27,28^. Consistently, our study on NER and DMT showed that they triggered protein damage at 2 h, while the inhibition of EGFR and AKT as well as the induction of DNA damage became obvious at 6 h (Fig. 4j). Next, we investigated the PDR and DNA damage response (DDR) induced by different drugs. Doxorubicin (DOB) and CIS induced DNA damage at 6 h as indicated by comet assay, and DNA damage was not observed within 12 h after LAP and gefitinib (GEF) treatment (Fig. 4k), while all 4 drugs induced strong protein damage at 1 h post treatment (Supplementary Fig. S1a). Consistently, DOB and CIS induced DDR at 6 h as indicated by phosphorylation of CHK1/CHK2 and upregulation of p21, whereas LAP and GEF did not induce DDR (Fig. 4l). Notably, all 4 drugs induced PDR at 1 h post treatment as indicated by the K48 polyUB incorporation (Fig. 4l). Thus, our finding shows that a vast majority of anticancer drugs damage proteins prior to reaching their canonical targets and trigger the PDR, which includes the ubiquitination and clearance of the damaged proteins.

### Inhibition of the PDR triggers cell death

**Fig. 5:**
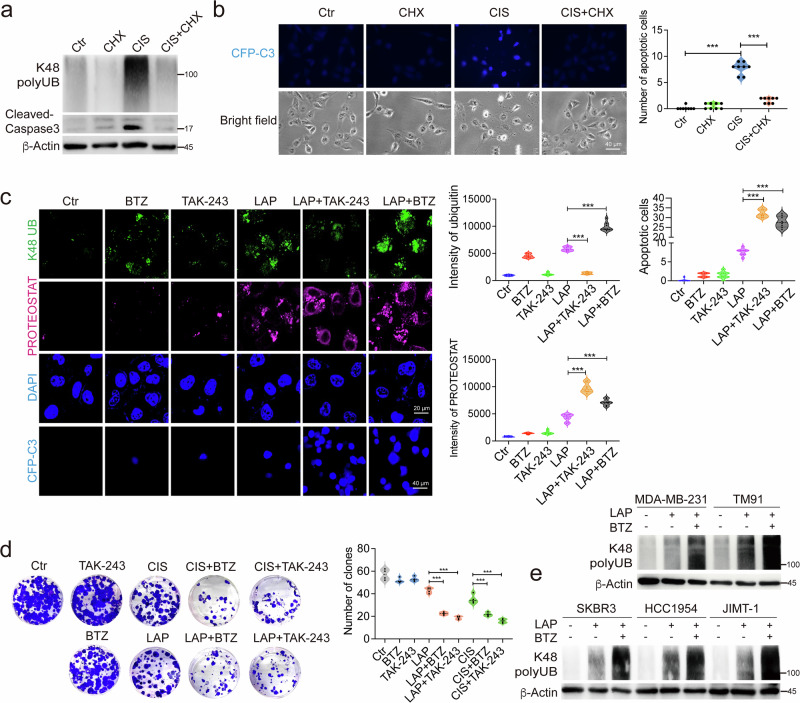

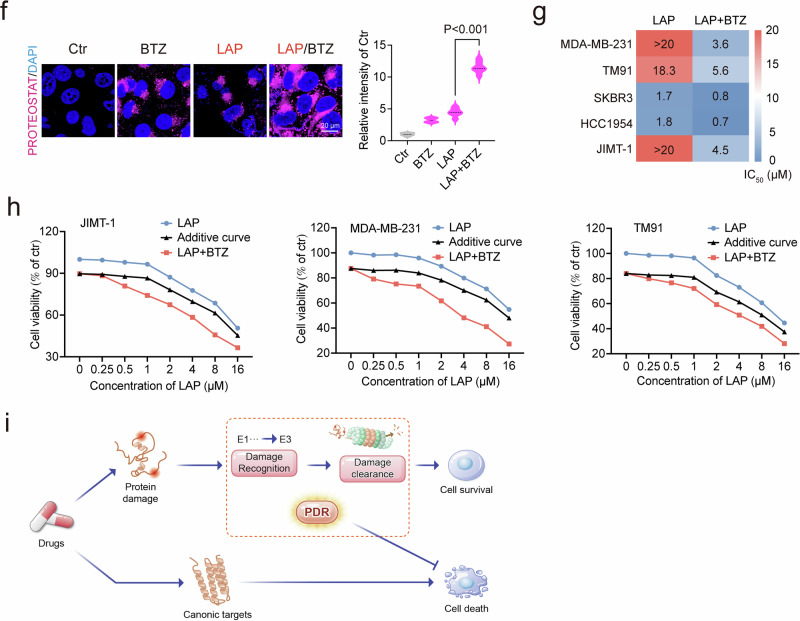
Inhibition of the PDR triggers cell death. **a** MDA-MB-231 cells were treated with CIS alone or in combination with CHX, and K48 polyUB and cleaved caspase 3 were detected by western blotting. **b** MDA-MB-231-C3 cells were treated with CIS alone or in combination with CHX, and caspase 3 activation was monitored by CFP fluorescence. **c** MDA-MB-231 cells were treated with LAP alone or in combination with BTZ or TAK-243, and K48 polyUB and protein aggregation were detected by immunofluorescence and PROTEOSTAT staining, respectively. MDA-MB-231-C3 cells were treated with LAP alone or in combination with BTZ or TAK-243, and caspase 3 activation was monitored by CFP fluorescence. **d** Representative Giemsa staining was used to observe clonogenic cell growth in 4T1 cells treated with indicated drugs, either alone or in combination with BTZ or TAK-243, and clones were quantified. **e** Five breast cancer cell lines were treated with LAP alone or in combination with BTZ treatment, and K48 polyUB was detected by western blotting. **f** MDA-MB-231 cells were treated with indicated drugs; representative images and quantification of PROTEOSTAT were shown. **g**, **h** Five breast cancer cell lines were treated with increasing doses of LAP alone or in combination with a single dose of BTZ, achieving 10%–16% killing effect in different cell lines, and cell viability was detected by alamar blue assay. IC_50_ of LAP treatment and LAP + BTZ treatment for 5 cell lines were calculated (**g**). Additive curves for LAP-resistant cell lines were shown, and additivity = E1 + E2 – E1 × E2, where E1 is the inhibitory effect of LAP and E2 is that of BTZ treatment (**h**). **i** A summary of the PDR. All values are presented as mean value (at least three replicates) ± SD, and *P* value was calculated by comparison with the Ctr group or indicated separately (two-tailed Student’s *t*-test, *P < 0.05, **P < 0.01, ***P < 0.001).

It is well established that most anticancer drugs exert their cytotoxic effects through specific cellular targets. However, since these drugs can induce protein damage prior to engaging their canonical targets, we sought to explore the potential role of protein damage in the cytotoxicity of anticancer drugs. To assess this, we utilized CHX treatment to inhibit protein damage and monitored the dynamics of caspase 3 activation using a fluorescence resonance energy transfer (FRET)-based C3 biosensor system^29^ (Supplementary Fig. S5a). Our findings revealed that CIS-induced protein damage was effectively mitigated by CHX treatment (Fig. 2g, h), which resulted in the inhibition of caspase 3 activation (Fig. 5a, b). These results suggest that drug-induced protein damage is a critical trigger for cell death. Next, we investigated the proteasome-dependent PDR and its pivotal role in managing protein damage induced by anticancer drugs. To illustrate this, we selected two representative drugs: CIS, which induces both protein and DNA damage, and LAP, which only causes protein damage. We then blocked protein damage recognition using TAK-243 treatment and protein damage clearance with BTZ treatment. Our data revealed that TAK-243 significantly reduced the levels of K48 polyUB induced by both LAP (Fig. 5c) and CIS (Supplementary Fig. S5b), while simultaneously leading to an increase in protein aggregation. This suggests that impaired recognition of damaged proteins resulted in further accumulation of them. Subsequently, we inhibited proteasome activity using BTZ to block the clearance of damaged proteins. The results demonstrated that BTZ treatment led to an accumulation of K48 polyUB, which corresponded with a marked increase in protein aggregation (Fig. 5c and Supplementary Fig. S5b). This indicates that while damaged proteins were recognized, they could not be effectively cleared from the cellular environment. Given the pivotal role of the PDR in managing protein damage accumulation, inhibition of the PDR should accelerate cell death. Indeed, the dramatic increase in protein aggregation was associated with increased cell death (Fig. 5c and Supplementary Fig. S5b). Similar results were observed in AXI-treated 4T1 cells (Supplementary Fig. S5c). To further demonstrate this, we assessed the colony formation ability of 4T1 cells. The results indicated that inhibition of either the protein damage recognition or clearance process was accompanied by a significant reduction in colony formation (Fig. 5d). Collectively, these data indicate that proteasome-dependent PDR acts as a protective response; inhibition of either damage recognition which is mediated by the ubiquitination system or damage clearance mediated by proteasomes markedly increases cytotoxicity.

To explore the relationship between the PDR and drug resistance, we investigated LAP, a therapeutic agent used in the treatment of HER2-positive breast cancers, where resistance frequently emerges^30^. We employed five breast cancer cell lines in this study: two HER2-negative (MDA-MB-231 and TM91) and three HER2-positive (SKBR3, HCC1954, and JIMT-1) (Supplementary Fig. S5d). LAP treatment was shown to induce protein damage, as evidenced by K48-mediated ubiquitination and protein aggregation, effects that were further enhanced by BTZ across all cell lines (Fig. 5e, f). Our findings indicated that, in general, HER2-negative cell lines exhibited greater resistance to LAP compared to HER2-positive cell lines, with the exception of JIMT-1 cells, which express HER2 at moderate levels and demonstrated high resistance to LAP (Fig. 5g). The addition of BTZ produced a synergistic effect across all five cell lines (Fig. 5g, h and Supplementary Fig. S5e), although this effect was less pronounced in SKBR3 and HCC1954 cells, which already showed a robust response to LAP alone (Supplementary Fig. S5e). These data suggest that, beyond targeting their canonical pathways, many anticancer drugs induce cell death by triggering protein damage. In response to this damage, cells activate the PDR, characterized by rapid ubiquitination of damaged proteins via the E1–3 system, followed by clearance through the proteasome, thereby promoting cell survival (Fig. 5i). This mechanism elucidates why inhibiting the PDR — through the use of E1 ubiquitin-activating enzyme inhibitors or proteasome inhibitors — can lead to the accumulation of protein damage and subsequently accelerate the cell death.

### Proteasome activity is associated with malignancy and serves as a prediction marker for MDR

**Fig. 6:**
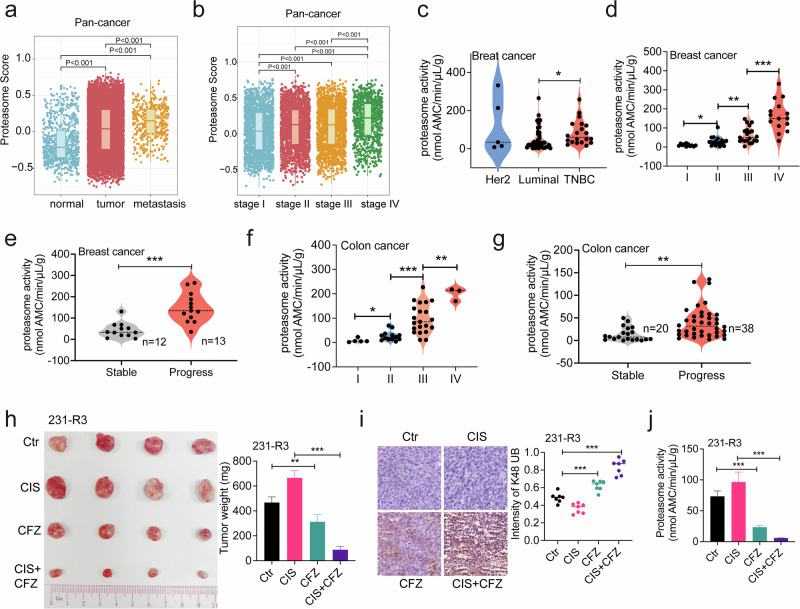

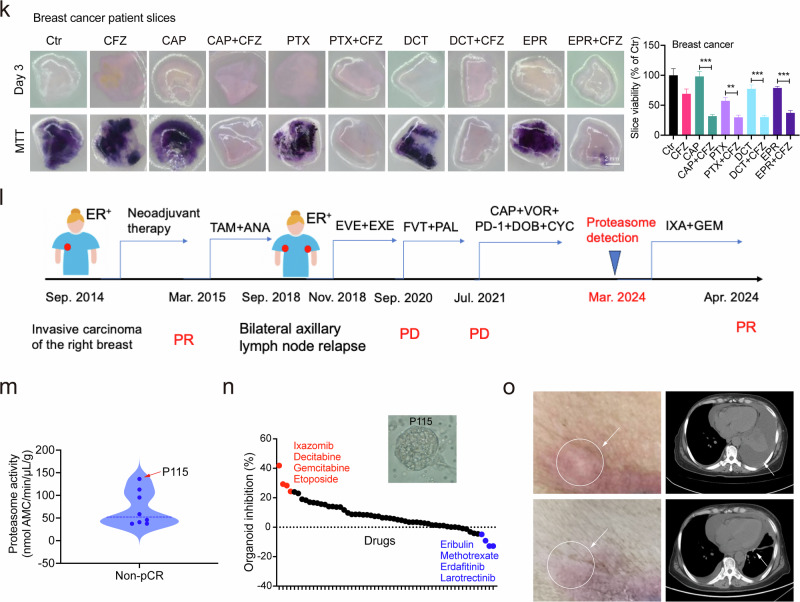
Proteasome activity is associated with malignancy and serves as a predictive marker for MDR. **a**, **b** Pan-cancer data from TCGA and TARGET comparing the proteasome activity scores (expression levels of proteasome genes, *n* = 45) in indicated tissues (**a**) or tumor stages (**b**). **c**, **d** The proteasome activity for breast cancer patients (*n* = 65) was detected by our kit, and proteasome activities in indicated breast cancer subtypes or tumor stages were compared. **e** The comparison of proteasome activities in resistant patients (PD) and sensitive patients (PR and SD) with breast cancer (*n* = 25). Stable and progressive disease following chemotherapy were evaluated based on the changes in tumor burden according to the RECIST guideline (v1.1). **f** The proteasome activity for colon cancer patients (*n* = 42) was detected by our kit and compared between indicated tumor stages. **g** The comparison of proteasome activities in resistant patients (PD) and sensitive patients (PR and SD) with colon cancer (*n* = 58). **h**–**j** Nude mice bearing 231-R3 multidrug-resistant cells were treated with vehicle, CIS (3 mg/kg) or CIS plus CFZ (1 mg/kg) every 3 days for 7 times, and tumor photograph and tumor weight were recorded (**h**). K48 polyUB in tumors was detected by IHC staining (**i**), and proteasome activity in tumors were detected by our kit (**j**). **k** Tumor slices were prepared from a breast cancer patient at stage IV and treated with the indicated drugs alone or in combination with CFZ. Slice viability was assessed by MTT staining. **l** Treatment procedure and responses of a breast cancer patient from surgery through proteasome activity assessment, followed by treatment with a proteasome inhibitor IXA. **m**, **n** Proteasome activity was tested in eight patients who did not achieve pathological complete response (non-pCR) (**m**); and the patient with the highest proteasome activity was sampled for PDO culture and screening of 52 drugs (**n**). **o** Tumor CT scan images and photos of the tumor site show that the malignant ascites and the tumor were shrunken during the GEM plus IXA treatment. All values are presented as mean value (at least three replicates) ± SD, and *P* value was calculated by comparison with the Ctr group or indicated separately (two-tailed Student’s *t*-test, *P < 0.05, **P < 0.01, ***P < 0.001).

To further investigate the relationship between drug resistance and protein damage clearance, we investigated the proteasome activity in the pan-cancer dataset encompassing over 10,000 samples spanning 40 cancer types, which were aggregated from The Cancer Genome Atlas (TCGA) and Therapeutically Applicable Research to Generate Effective Treatments (TARGET) databases. The data indicated that the proteasome activity score was higher in the tumor and further increased in the more malignant metastasis (Fig. 6a), implying that the proteasome activity is associated with the tumor malignancy. Indeed, with tumor progression, the proteasome activity score gradually increased in the pan-cancer patients (Fig. 6b). These results and our data indicated that proteasome activity should be a key marker for drug resistance. To identify potential drug-resistant patients, we developed a highly sensitive proteasome activity detection kit that can detect micro biopsy samples in clinical settings. Using this kit, we then investigated 65 breast cancer patients for their proteasome activity. The results indicate that the proteasome activity is the highest in the TNBC patients, which is more malignant than luminal and Her2-positive patients (Fig. 6c). Similarly, the proteasome activity was found to be increasing from tumor stage I to IV, indicating that proteasome activity is associated with tumor malignancy (Fig. 6d). We then followed 25 breast cancer patients who received various drug treatments to assess their responses to therapy and their proteasome activity (Supplementary Fig. S6a). Our data indicated significant higher proteasome activity in patients who exhibited progressive disease (PD, *n* = 13) than patients who displayed partial response (PR, *n* = 9) or stable disease (SD, *n* = 3) (Fig. 6e). A patient with HER2-positive ductal carcinoma in situ on the left side underwent surgery after neoadjuvant chemotherapy in October 2016. The tumor relapsed and metastasized to the right breast in November 2019. She received 6 cycles of chemotherapy and HER2-targeted treatments but experienced PD. A proteasome activity test showed high levels (213.6 nmol AMC/min/µL/g), and subsequent treatments with Nolvadex, Herceptin, and antibody-drug conjugates also resulted in PD (Supplementary Fig. S6b).

In colon cancer patients, we tested proteasome activity in 42 individuals and found that it increased from tumor stage I to IV (Fig. 6f). Additionally, we followed 58 colon cancer patients, all at stage IV, who received various drug treatment regimens (Supplementary Fig. S6c). We detected higher proteasome activities in 38 patients who developed PD compared to 20 patients with PR or SD (Fig. 6g). Consistently, in the TCGA and GES databases, two cohorts of colon cancer patients treated with capecitabine and bevacizumab, respectively, showed that the resistant patients (non-responders) had significantly higher proteasome activity scores compared to sensitive patients (responders). Additionally, two other cancer types were identified in which resistant patients also exhibited elevated proteasome activity scores (Supplementary Fig. S6d).

To test whether inhibition of proteasome activity can overcome MDR, we utilized the xenograft of 231-R3 cells, a multidrug-resistant cell line developed through an evolutionary model^7^. Indeed, mice treated with CIS alone exhibited more aggressive tumor growth and drug resistance, which was significantly reversed by the combination with CFZ treatment (Fig. 6h). Notably, K48 polyUB was dramatically increased by CFZ treatment (Fig. 6i), which was accompanied by the suppression of proteasome activity in these tumors (Fig. 6j). These results indicate that inhibition of damaged protein clearance by proteasome inhibitor treatment further enhanced the protein damage and resulted in the reversal of drug resistance. To mitigate potential off-target effects of proteasome inhibitors, we used shRNA to target PSMB7 in MDA-MB-231 cells. Nude mice bearing MDA-MB-231 or MDA-MB-231-shPSMB7 cells were treated with CIS. The MDA-MB-231-shPSMB7 cells were more sensitive to CIS treatment, which is correlated with enhanced ubiquitination and increased cleaved caspase 3 in the tumor (Supplementary Fig. S6e, f).

Next, we utilized a three-dimensional tumor slice culture (3D-TSC)-based drug sensitivity testing platform^31^. We selected a stage IV breast cancer patient diagnosed with high proteasome activity (212.8 nmol AMC/min/µL/g) to prepare the 3D-TSC. Consistent with the fact that this patient did not respond to the capecitabine (CAP)/EPR treatment, the 3D-TSCs were also resistant to this treatment as well as to paclitaxel (PTX)/docetaxel (DCT) (Fig. 6k). When these drugs were combined with the BTZ treatment, the resistance was overcome for all 4 structurally unrelated anticancer agents as indicated by the much weaker MTT staining of the 3D-TSCs (Fig. 6k). Similar results were observed in a stage IV colon cancer patient (Supplementary Fig. S6g), indicating the potential of blocking proteasome activity to overcome MDR in proteasome-high patients.

Next, an investigator-initiated clinical trial was launched for eight breast cancer patients. P115 has been diagnosed with ER-positive breast cancer since September 2014, and neoadjuvant therapy initially achieved a PR (Fig. 6l). Four years later, the tumor relapsed, and multiple rounds of treatment consistently resulted in PD. Subsequently, in March 2024, P115 was sampled for proteasome activity detection. Among all eight patients tested, P115 exhibited the highest proteasome activity (136.3 nmol AMC/min/µL/g) and was selected for further investigation (Fig. 6m). Patient-derived organoids (PDOs) have proven to be reliable systems for cancer drug discovery and assessment^32^. We first established PDOs from P115 (Fig. 6n) and screened 57 anticancer drugs to identify the most promising candidate. Notably, ixazomib (IXA), a proteasome inhibitor, ranked first (Fig. 6n). Consequently, IXA was combined with GEM for 1 month of administration, resulting in a marked reduction of skin lesions (Fig. 6o). Additionally, the right lung, previously compressed by ascitic swelling, also showed significant improvement (Fig. 6o).

Altogether, these results suggest a model through which the majority of anticancer drug damage proteins prior to reaching their canonical targets, triggering the PDR. The PDR contains two major steps, i.e., the damage recognition, which is mainly mediated by the ubiquitin system, and the damage clearance, which is mainly mediated by proteasome system. Treatment with proteasome inhibitors could result in the accumulation of protein damage, leading to the accelerated cell death. Conversely, cells with higher levels of proteasome activity intrinsically or gradually forced to increase proteasome activity should survive better to the cytotoxicity of anticancer drugs, which might be a reason why the drug-resistant breast and colon cancers exhibit higher levels of proteasome activity compared to drug-responsive tumors (Fig. 6e, g).

## Discussion

MDR in cancer occurs very frequently and can be affected by many factors, including DNA damage repair, drug target mutation, drug metabolism and inactivation, cellular drug excretion, aberrant cell death regulation, and cancer stem cells^5,6^. In this study, we provide strong evidence that a vast majority of anticancer drugs damage proteins prior to reaching their canonical targets and induce PDR. Because PDR has been rarely recognized previously, we focused on its role in triggering MDR by counteracting protein damage induced by anticancer drugs, and on developing strategies to detect and suppress PDR.

There are several features of the protein damage and PDR caused by anticancer drugs. It has been known that some anticancer drugs can bind their target proteins, and many studies have emphasized on the relatively specific binding of the drugs to some specific domains, sites, structures^33–40^. However, we found that protein damage occurs rapidly and on a large scale, suggesting that the damage is caused by non-specific, rather than specific binding of these drugs to the proteins. Of note, the PDR is distinct but shares conceptual overlap with some other integrated stress response pathways, such as the unfold protein response (UPR) and ribosome-associated quality control (RQC). While the UPR functions as a general protein quality control mechanism within the cells specifically against misfolded proteins^41^, and the RQC monitors ribosomes for aberrant translation at the initiation, elongation, and termination steps^42^, the PDR mainly refers to protein damages caused by anticancer drug treatment, including not only misfolded proteins, but also structural abnormalities and oxidative damage. The PDR might affect protein synthesis, as we found that many of the damaged proteins are involved in ribosome (Fig. 1k and Supplementary Fig. S1e), leading to the activation of RQC. However, the relationship between RQC and PDR needs future investigation.

We also found that many of these drugs tested also damage DNA and induce DDR^43–45^, which is known as a protective cellular response to counteract the devastating effects associated with DNA damage^46,47^. Our data indicated that anticancer drugs damage proteins more extensively and promptly than they damage DNA. This is largely because neo-peptides and newly synthesized proteins are in the cytoplasm whereas DNA is in the nucleus. Upon entering the cell, drugs first reach protein, and then after a longer treatment, some drugs also cause DNA damage. PDR is a protective response of cells to remove damaged proteins and avoid cytotoxicity, as the blockade of PDR results in the accumulation of elevated protein damage and the accelerated cell death.

We demonstrated that the PDR contains two major steps, i.e., the damage recognition, which is mainly mediated by ubiquitin system, and damage clearance, which is mainly mediated by proteasome systems, with the involvement of some other factors, such as ROS and cell proliferation. Ubiquitination is normally required by cells for post-translational modification in which ubiquitin is conjugated as a single ubiquitin (monoubiquitination) or a polyubiquitination chain to a protein sequentially by the E1 (activating)-E2 (conjugating)-E3 (ligating) enzyme cascade^48–50^. Our data indicate that although there are massive and quick ubiquitination of damaged proteins, the use of TAK-243, an inhibitor of E1 enzyme activity^25^ could completely suppress the ubiquitination, highlighting a role of the E1-E2-E3 ubiquitin system in this step. Ubiquitinated proteins are subjected to degradation by the proteasome systems. Consistently, our data indicated that bortezomib markedly accumulated damaged proteins and increased cell death. Bortezomib is the first proteasome inhibitor approved by the U.S. FDA, followed by next-generation protein inhibitors, carfilzomib (CFZ) and IXA^51,52^.

Acquired drug resistance commonly occurs during and after treatment through various mechanisms^5^. To identify genes that may be responsible for this acquired drug resistance, we previously used RNAi libraries with increasing sizes to conduct screens. Our first RNAi library contained 56 genes, from which we identified ATP7A, which excretes cisplatin from cells, as a potential target that confers cisplatin resistance^10^. We then screened an RNAi library containing 706 kinases and identified ATR, CHK1 and WEE1, which shut down DNA replication and attenuate cisplatin-induced lethality^11^. While these screens confirmed that many factors may be involved in drug resistance, they were limited to identifying the most potent mechanisms underlying the generation of drug resistance because of the small number of genes screened. Thus, to identify the strongest molecular basis underlying cisplatin resistance, we finally applied whole-genome RNAi screening together with tumor drug resistance evolution models and found that cisplatin resistance is closely related to the ability of cancer cells to remove damaged proteins^7^. In proliferating cells, drug treatment induces extensive protein damage, resulting in strong cytotoxicity. On the other hand, because 99% of mitochondrial proteins are imported from the cytoplasm^53^, the impairment of mitochondrial OXPHOS and shut down of ATP production due to these damaged proteins are unavoidable, which not only reduces new protein synthesis but also activates proteasome activity to reduce the loading of damaged proteins and enable cells to gradually acquire drug resistance.

Cancer cells, in general, divide faster than the cells in normal tissues and organs, and many cancers usually occur more frequently in older individuals, in which a vast majority of noncancerous cells are less proliferative^54,55^. We also showed that our strategy is less toxic to healthy tissues due to their decreased degree of cell division and the presence of low baseline proteotoxic stress. Thus, targeting the damaged protein clearance pathway with our combination drug regimen serves as a promising and safe strategy to eliminate refractory cancer cells.

In summary, our data indicate that a majority of anticancer drugs kill cells by damaging newly synthesized proteins prior to affecting their canonical targets. At the same time, cancer cells initiate PDR to minimize the cytotoxicity as a mechanism to combat these drugs. The PDR includes protein damage recognition and clearance with the involvement of some other factors, such as ROS, protein synthesis rate and mitochondrial respiratory activities, enabling them to acquire MDR (Supplementary Fig. S7). Because MDR is a major problem and kills the majority of cancer patients^4–6^, we developed a sensitive kit for detecting proteasome activity, which is a key component for PDR. Our study may provide a promising strategy to determine the drug resistance status of cancer patients before, during and after treatment by measuring their proteasome activity, and thus, proper therapeutic options may be designed accordingly.

## Materials and methods

### Animals and housing conditions

Mice were housed in a specific-pathogen-free (SPF) facility at 23–25 °C on a 12-h light/dark cycle. The mice were fed a standard rodent diet (PicoLab Rodent 5053 Laboratory Diet). Nude mice (5 weeks) were used in this study.

### Ethical statement

All animal procedures were conducted under the ethical guidelines of the University of Macau (animal protocol number: UMARE-AMEND-219), ensuring that all experiments adhered to ethical standards for the care and use of laboratory animals.

### Allograft/xenograft studies

MDA-MB-231 and 231-R3 multidrug-resistant cells (derived from MDA-MB-231 cells) were cultured in DMEM with 10% fetal bovine serum. The cells were trypsinized and resuspended in PBS. Then, the cells were mixed with Matrigel (Corning, 354234) at a 1:1 (v/v) ratio, and 1.0 × 10^6^ cells were injected subcutaneously into the flanks of nude mice. One mouse bearing MDA-MB-231 cells was treated with cisplatin (3 mg/kg) and tumor samples were collected by little biopsy at 0, 24, 48, and 72 h, respectively; and the other mouse bearing MDA-MB-231 cells was treated with cisplatin (3 mg/kg) plus with BTZ (2 mg/kg), and samples were collected by little biopsy at 72 h. Samples were analyzed for K48 Ub and γH_2_AX by western blotting.

Nude mice bearing 231-R3 multidrug-resistant cells began receiving drug treatment when the tumor volume reached ~200 mm^3^. The mice were treated with cisplatin (3 mg/kg) alone or in combination with CFZ (1 mg/kg) by intraperitoneal injection for seven cycles, with an interval of 3 days between each treatment.

### Immunohistochemical (IHC) staining

Routine haematoxylin staining of 6-μm sections of formalin-fixed, paraffin-embedded (FFPE) tissues was performed. IHC staining for K48 Ub (1:100, cell signaling technology, #4289) was performed by a Histostain-Plus IHC Kit (Thermo Fisher Scientific, 859043, 1954379A) after antigen retrieval (pH 6.0), quenching of endogenous peroxidase activity and overnight incubation with the primary antibody.

### Cell culture

T47D, MCF7, MDA-MB-231, HepG2, A549, HCT, TM91, JIMT-1, HCC1954, and SKBR3 cells were cultured in DMEM supplemented with 10% fetal bovine serum, glutamine (Thermo Fisher Scientific), and pen/strep. The cisplatin-resistant cell lines 231-R1, 231-R2, and 231-R3 were established as previously described^7^.

### 3D-TSCs

3D-TSCs were prepared from allograft and xenograft tumors. Thick tissue slices (200 μm) were prepared under sterile conditions using a Leica VT1200 S vibratome (Leica Biosystems Nussloch GmbH, Germany) within 2 h after surgery. An embedding collagen mixture containing rat collagen I, 10× Ham’s F-12 medium, and sterile reconstitution buffer (2.2 g NaHCO_3_ in 100 mL of 0.05 N NaOH and 200 mM HEPES) at a ratio of 8:1:1 was prepared. The thick tissue slices were embedded in 100 μL of the collagen mixture on the bottom and 50 μL of the collagen mixture on the top. The slices were then placed on 0.4-mm pore-size membrane culture inserts in 24-well plates and cultured in F medium, which contained DMEM/F12 (Thermo Fisher Scientific), 5% FBS, 10 ng/mL EGF (Thermo Fisher Scientific, 13247-051), 0.5 mg/mL hydrocortisone (Sigma, H0888), 20 ng/mL cholera toxin (Sigma, C-3012), 5 μg/mL insulin (Sigma, 350-020), 300 U/mL collagenase III (Worthington, S4M7602S) and hyaluronidase (Sigma, H3506). Drug treatment was performed after overnight culture, and images were taken with a Leica M165FC fluorescence stereomicroscope. For MTT staining, the dye MTT (3-[4,5-dimethylthiazol-2-yl]-2,5-diphenyltetrazolium bromide; thiazolyl blue, Sigma-Aldrich) was added (final concentration: 0.5 mg/mL) to tissue slices and incubated for 4 h. The formazan crystals were solubilized with DMSO, and the absorbance at 570 nm was measured using a plate spectrophotometer.

### Chemicals and anticancer drug library

The anticancer drug library used to screen for protein damage and DNA damage and chemicals used in this study are summarized in Supplementary Table S1. Other chemicals include Puro (InvivoGen, ant-pr-1), CHX (biorbyt, orb340214a), salubrinal (Selleckchem, S2923-5mg), NAC (Beyotime, S0077), thymidine (Sigma-Aldrich, T9250), L-ascorbic acid (Beyotime, ST1434-25g), CFZ (MedChemExpress, HY-10455/50 mg), BTZ (MedChemExpress, HY-10227/100 mg), IXA (MedChemExpress, HY-10453/25 mg), Dox (Sigma-Aldrich, D9891), 2,2-dichloroacetophenone (Sigma-Aldrich, D54850), biotin (MedChemExpress, HY-B0511), CIS-biotin (Xi’an ruixi Biological Technology Co., Ltd, R-HGF148), and streptavidin agarose (Sigma-Aldrich, 85881).

### Cell viability analysis

Cell viability was determined by the alamar blue assay. Briefly, cultured cells were plated in a 96-well plate at 5000 cells per well and cultured overnight. The cells were treated with the indicated drugs for 48 h. Then, the cells were incubated with medium containing 0.02% alamar blue for 2 h, and cell viability was determined by measuring the absorbance at 590 nm under 560-nm excitation.

### TCGA/TARGET database analysis

The present study leveraged a pan-cancer dataset from the TCGA and TARGET databases. Upper-quartile normalized FPKM RNA-sequencing data were retrieved from the UCSC XENA resource^56^, while clinical data were acquired through the R package UCSCXenaTools. Subsequently, proteasome activity score was quantified for each sample using the Gene Set Variation Analysis (GSVA) function implemented in the gsva R package^57^.

### Western blotting and immunoprecipitation

Cultured cells were homogenized in RIPA buffer supplemented with phosphatase and protease inhibitor cocktails (Sigma). The protein concentration was detected by a BCA protein assay kit, and samples containing equivalent protein concentrations were resolved by SDS/PAGE (10% gels). The proteins were then transferred to PVDF membranes (Bio-Rad), which were blocked with 5% BSA in TBS-T buffer for 1 h at room temperature and incubated with primary antibody overnight at 4 °C. The PVDF membranes were incubated with the corresponding secondary antibodies (Cell Signaling Technology, CST), and the protein bands were visualized with horseradish peroxidase substrate (Millipore) under a ChemiDoc Touch Imaging System (Bio-Rad). Primary antibodies against the following were used in this study: ubiquitin (1:1000, CST, 3936S), γH_2_AX (1:1000, CST, 9718S), β-actin (1:1000, CST, 3700S), HA (1:2000, Sigma-Aldrich, H6908), Myc (1:200, Santa Cruz Biotechnology, sc-40), FLAG (1:2000, Sigma-Aldrich, F1804), cleaved caspase 3 (1:1000, CST, 9664S), anti-Puro (1:1000, Merck, MABE343), GAPDH (1:1000, CST, 5174S), GFP (1:200, Santa Cruz Biotechnology, sc-9996), luciferase (1:200, Santa Cruz Biotechnology, sc-57603), p4E-BP1 (1:1000, CST, 9451), 4E-BP1 (1:1000, CST, 9452), p70S6K (1:200, Merck Millipore, 06-926), p-p70S6K (1:1000, CST, 9234), pAKT (1:1000, CST, 9271), mTOR (1:1000, CST, 2983), and p-mTOR (1:1000, CST, 5536).

For immunoprecipitation, cultured cells were homogenized in IP cell lysis buffer (Beyotime) and incubated with primary antibody and Protein A/G PLUS-Agarose (Santa Cruz Biotechnology, sc-2003) overnight at 4 °C. Proteins of interest were then pulled down by centrifugation (Eppendorf) and detected by western blotting.

### Protein aggregation detection assay

The PROTEOSTAT aggresome detection kit (Enzo Life Sciences, ENZ-51035-K100) was used to detect mis-folded or aggregated proteins in cells treated with indicated anticancer drugs. The PROTEOSTAT staining was performed according to the manufacturer’s instructions. Briefly, MDA-MB-231 cells were treated with indicated drugs, fixed, and permeabilized. The PROTEOSTAT was observed by 488 excitation and 600–620 emission on a Carl Zeiss LSM880 confocal microscope.

### Protein carbonyl detection

Cultured cells were lysed, and proteins were extracted and processed using a Protein Carbonyl Content Assay Kit (Abcam, ab178020) according to the manufacturer’s instructions. Briefly, the carbonyl groups in the protein side chains were derivatized to 2,4-dinitrophenylhydrazone (DNP-hydrazone) by reaction with 2,4-dinitrophenylhydrazine (DNPH), and DNP-hydrazone was detected by western blotting with primary anti-DNP antibody.

### Proteasome activity detection

Patient tumor tissues were homogenized in 0.5% NP40, and proteasome activity was measured by our kit. Briefly, tissue lysates were incubated with Suc-Leu-Leu-Val-Tyr-7-amino-4-methylcoumarin (Succ-LLVY-AMC), and the free AMC fluorescence was measured at 350/440 nm. The increase in AMC fluorescence (ΔRFU) was detected as proteasomal activity as follows: ΔRFU = (RFU2 – iRFU2) – (RFU1 – iRFU1), where RFUn is the total proteolytic activation at time n and iRFUn is the non-proteasomal proteolytic activity at time n. Our assay demonstrates significantly enhanced sensitivity compared to commercially available kits. We observed a 7.5-fold increase in signal following substrate degradation, indicating superior detection capabilities. Furthermore, our assay achieves a lower limit of detection of 8000 cells, a substantial improvement over the 200,000-cell limit of commercial alternatives. This enhanced performance is primarily attributed to the optimized composition of our buffer solution and the replacement of a specific proteasome inhibitor with BTZ. Further details and supporting data can be found in patent ZL 2022 1 1119256.5.

### Firefly luciferase activity test

The luciferase activity was detected using the Rapid Detection of Firefly Luciferase Activity Kit (Promega, E1500) according to the manufactory’s instruction. Briefly, 1× lysis reagent was dispensed into each culture vessel. Cell lysis was centrifuged, and the supernatant was transferred to a new tube. Then, 20 μL of cell lysate and 100 μL of Luciferase Assay Reagent were mixed in the tube, and the luciferase activity was measured based on the emitted luminescence.

### Proteomics for CIS-biotin binding assay

MDA-MB-231 cells were treated with 50 μM biotin, 50 μM CIS-biotin, 50 μM CIS-biotin + 20 nM BTZ, or 50 μM CIS-biotin + 50 µg/mL CHX for 3 h and then lysed. Biotin was pull down by the streptavidin immobilized on the agarose beads. The binding proteins were analyzed by LC-MS/MS. The protein peak data were utilized to represent the final expression levels of a specific protein across various samples, resulting in a 5106 × 9 and 3751 × 12 protein-expression matrix for biotin treatment, respectively. Missing values were imputed as one-tenth of the minimum value across our proteome dataset. The Student’s *t*-test, implemented in R software, was used to identify proteins differentially expressed between the following groups: CIS-biotin vs Biotin, CIS-biotin + CHX vs Biotin, and CIS-biotin + BTZ vs Biotin. Proteins were considered upregulated if they were differentially expressed compared to the Ctr or Biotin group (fold change (expressed as log_2_(ratio of average protein abundance)) > 1, *P* < 0.05).

### LiP-MS assay

The Lip-MS assay was conducted as previously reported^17^. Briefly, the MDA-MB-231 cells were treated with 50 µM CIS for 1 h, after which the cells were lysed. Half of the sample underwent limited proteolysis (LiP) with proteinase K (Promega, V3021) for 1 min at 25 °C. The proteinase K was then inactivated by boiling the sample in water. This was followed by incubation with deoxycholic acid (DOC, sigmaaldrich-D6750) to a final concentration of 5% (w/v). The proteinase K-digested samples were processed, while the remaining half of the sample underwent conventional digestion with LysC (Thermos Fisher Scientific, 90307) and trypsin (Promega, V5111). The resulting peptides were purified using C18 Spin Tips (Thermo Fisher Scientific, 89870-25). All samples were analyzed by conventional LC-MS/MS, and the peptide matrix was evaluated according to a previous report for the identification of differential peptide patterns^17^.

### Ubiquitin-modified proteome

The ubiquitin-modified proteome analysis was conducted by Qinglianbio (Beijing, China). Briefly, MDA-MB-231 cells were treated with either vehicle or 50 µM CIS for 6 h. Approximately 5 × 10^7^ cells were required for each sample, and three biological replicates were prepared for both the vehicle and CIS-treated groups. The samples were digested with trypsin and underwent affinity enrichment using an anti-Lys-ε-Gly-Gly (K-ε-GG) antibody, as previously reported^58^. The resulting samples were analyzed by LC-MS/MS. The MS/MS data were processed using the MaxQuant search engine (v1.5.2.8)

### CRISPR Cas9 sgRNA and shRNA transfection

sgRNAs were cloned and inserted into the lentiviral vector TLCV2 (Addgene Plasmid #87360) according to the manufacturer’s instructions. After transfection, positive cells were selected by Puro (Thermo Fisher Scientific). The following sequence was used: PSMB5, CAAGTCCGAAAAACCCGCGC. The shRNA targeting PSMB7 (AATGGCTGTATTTGAAGATAA) was cloned into the pLKO.1-TRC vector (Addgene, #10878). The knockdown and knockout effects were validated by qPCR.

### Human specimens and clinical case study

This study involved the assessment of proteasome activity in breast cancer and colon cancer specimens collected from three hospitals in China. Ethical approval was obtained from the ethics committees of each institution, including The First Affiliated Hospital of Sun Yat-sen University, Sun Yat-sen University Cancer Center, and The Seventh Affiliated Hospital of Sun Yat-sen University. Informed consent was secured from all donors prior to sample collection. Clinical information was gathered from the medical record systems. Small biopsy samples from tumor tissues were homogenized, and proteasome activity was detected using our kit.

The clinical case study is associated with an investigator-initiated clinical trial investigating the drug IXA. The samples obtained from patients were tested for proteasome activity and used for the development of PDOs, and the study was assessed and approved by the respective ethics committees. All patients provided written informed consent in accordance with the principles of the Declaration of Helsinki.

### Figure construction

Graphs were created in Prism (v9) and assembled into figures in Adobe Illustrator 2022. All experiments were conducted at least three times independently. Microsoft Excel and Prism (v9) were used for statistical calculations, and *t*-test analysis was used to determine the significance of the difference between different sets of data.

## Supplementary information

Supplementary Information

## Data Availability

The datasets used and/or analyzed in the current study are available in Supplementary Table S2, and further information is available from the corresponding author upon reasonable request.
