# Supplementary material for: Suppressing protein damage response to overcome multidrug resistance in cancer therapy
Source: Cell Discov. 2025 Sep 30;11:80. doi: 10.1038/s41421-025-00826-9 (PMC12484725; doi:10.1038/s41421-025-00826-9)
Supplement: Supplementary file 1 — Supplementary Information [file 41421_2025_826_MOESM1_ESM.pdf]

Supplementary Figures

a

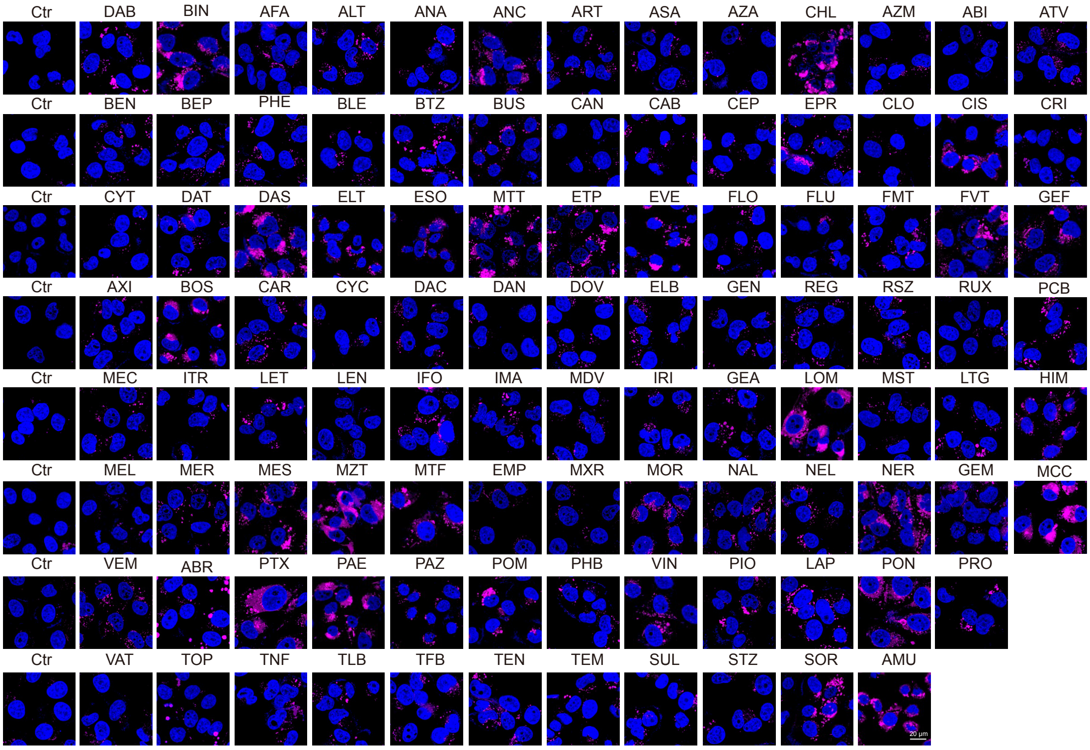

b

Compound	Target	Type	Compound	Target	Type	Compound	Target	Type
Anti-inflammatory/Pain Relief			DNA/RNA Synthesis			Topoisomerase		
Phenylbutazone	COX-1/2	***	Ancitabine	Nucleoside analog	**	Etoposide	Topoisomerase II	***
Aspirin	COX-1/2	**	Carmofur	Thymidylate Synthase	**	Teniposide	Topoisomerase II	***
Sulindac	COX-1/2	**	Floxuridine	Pyrimidine analog	**	Irinotecan	Topoisomerase I	**
Bepotastine	Histamine receptor	**	Gemcitabine	Cytidine analog	**	Topotecan	Topoisomerase I	**
Geniposidic	NA.	*	Mercaptopurine	Purine analog	**	Epirubicin	Topoisomerase II/DNA	*
Tyrosine kinase inhibitor			Nelarabine	DNA polymerase	**	Mitoxantrone	Topoisomerase II/DNA	*
Amuvatinib	FLT3	***	Azaguanine-8	Purine analog	ns.	Cytoskeleton		
Bosutinib	BCR-ABL	***	Abitrexate	DHFR	ns.	10-Deacetylbaccatin	Microtubule	**
Dasatinib	BCR-ABL	***	Clofarabine	Purine analog	ns.	Cephalomannine	Microtubule	*
Gefitinib	EGFR	***	Cytarabine	Pyrimidine analog	ns.	Paclitaxel	Microtubule	*
Lapatinib	HER2	***	Fludarabine	Purine analog	ns.	Vincristine	Microtubule	*
Neratinib	HER2	***	Antibiotic			Others		
Sorafenib	VEGFR/PDGFR/BRAF	***	Methacycline	Bacterial ribosome	***	Bortezomib	Proteasome	***
Ponatinib	BCR-ABL/VEGFR/FGFR	***	Azithromycin	Bacterial ribosome	*	DAPT	γ-secretase	***
Tofacitinib	JAK1/3	***	Tolnaftate	Antifungal	**	Eltrombopag	c-Mpl receptor	***
Afatinib	EGFR	**	Itraconazole	Antifungal	*	Methazolastone	NA.	***
Dovitinib	VEGFR	**	Antiviral			Mitotane	NA.	***
Erlotinib	EGFR	**	Moroxydine	Protein synthesis	***	Paeoniflorin	NA.	***
Imatinib	BCR-ABL	**	Atazanavir	protease enzyme	**	Artemether	ROS	**
Regorafenib	VEGFR/PDGFR/c-KIT	**	DNA binding			Esomeprazole	Proton pump inhibitor	**
Pazopanib	VEGFR/PDGFR/c-KIT	**	Bindarit	DNA	***	Everolimus	mTOR pathway	**
Vemurafenib	BRAF	**	Chlorambucil	DNA	***	Lamotrigine	Ion channel	**
Axitinib	VEGFR	*	Cisplatin	DNA	***	Miltefosine	Anti-parasitic	**
Crizotinib	ALK	*	Ifosfamide	DNA	***	Mesna	NA.	**
Masitinib	c-KIT/PDGFR/FGFR	*	Lomustine	DNA	***	Naloxone	NA.	**
Vatalanib	VEGFR/PDGFR	ns.	Procarbazine	DNA	***	Rosiglitazone	PPAR-γ	**
Ruxolitinib	JAK1/2	ns.	Dacarbazine	DNA	**	Temocapril	NA.	**
Androgen/estrogen sinaling			Altretamine	DNA	*	Tolbutamide	NA.	**
Formestane	Aromatase	**	Bendamustine	DNA	*	Phenylbutyric	HDAC	**
Letrozole	Aromatase	**	Bleomycin	DNA	*	Proadifen	NA.	*
Abiraterone	CYP17A1	*	Busulfan	DNA	*	Pomalidomide	NA.	*
Anastrozole	Aromatase	*	Carboplatin	DNA	*	Pioglitazone	PPAR-γ	*
Fulvestrant	Estrogen receptor	*	Mechlorethamine	DNA	*	Danusertib	Cell cycle	ns.
Genistein	Estrogen receptor	*	Streptozotocin	DNA	*	Cantharidin	PP2A	ns.
Mdv3100	Androgen receptor	*	Cyclophosphamide	DNA	ns.	Lenalidomide	NA.	ns.
Melengestrol	Progesterone receptors	ns.	Estramustine	DNA	ns.			

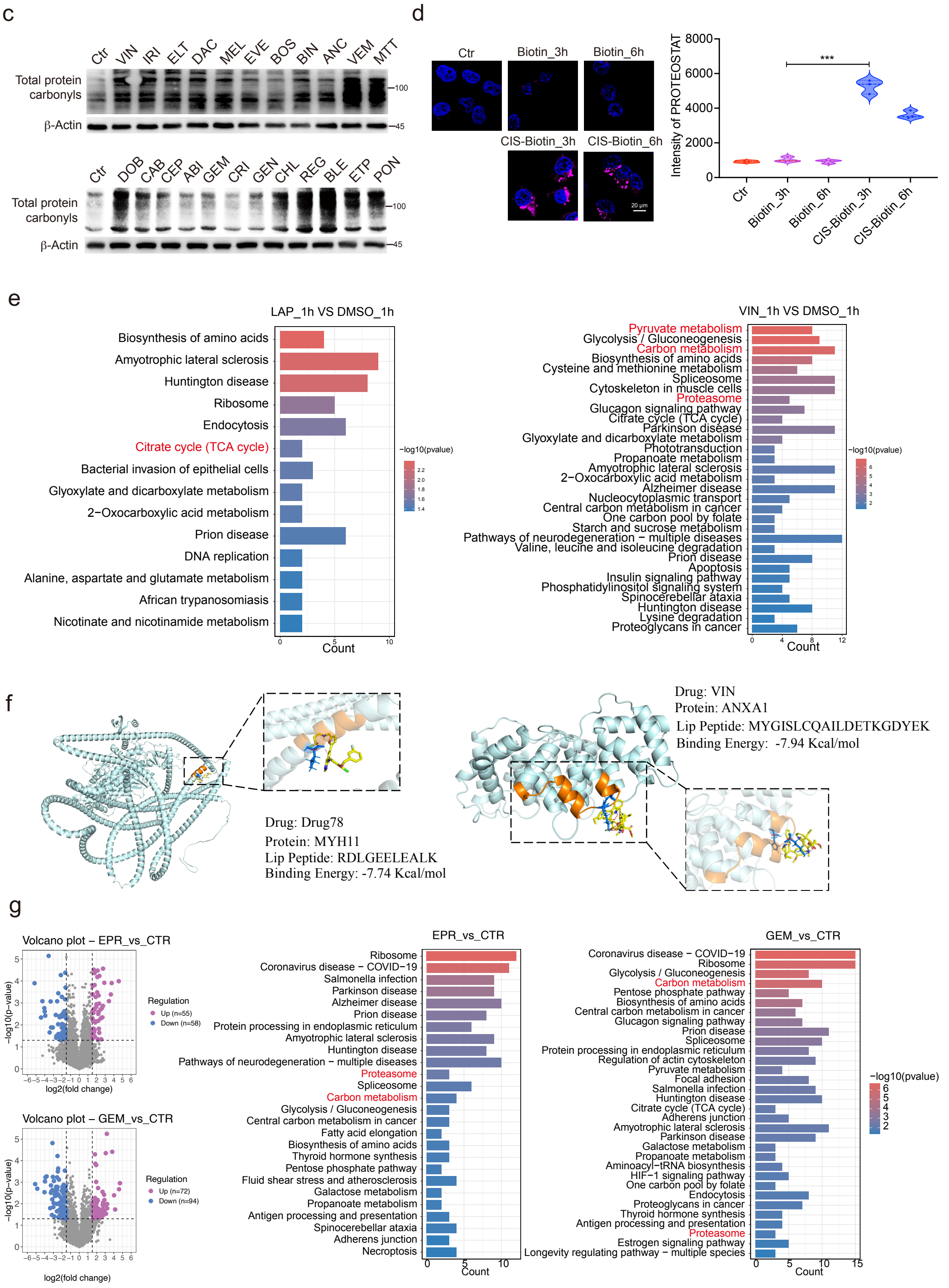

Supplementary Fig S1. The vast majority of anticancer drugs induce a wide range of protein damage. (A) MDA-MB-231 cells were treated with 101 drugs for 1 hour, and protein aggregation were stain by the PROTEOSTAT. (B) The working mechanism of 101 anticancer drugs and their types of protein damage. (C) 23 drugs were tested for protein oxidation damage. (D) MDA-MB-231 cells were treated with indicated drugs

and protein aggregation were stain by the PROTEOSTAT. (E) MDA-MB-231 cells were treated with *LAP* or *VIN* for 1 hour, and proteins with differential MS peptide profiling were identified and analyzed by KEGG enrichment. (F) PyMOL analysis of protein steric structure showing peptides identified by Lip-MS. Molecular docking predicted a *LAP or VIN* binding energy of -7.7 kcal/mol and 7.9 kcal/mol, respectively. (G) MDA-MB-231 cells were treated with *EPR* or *GEM* for 1 hour, and proteins with differential MS peptide profiling were identified and analyzed by KEGG enrichment.

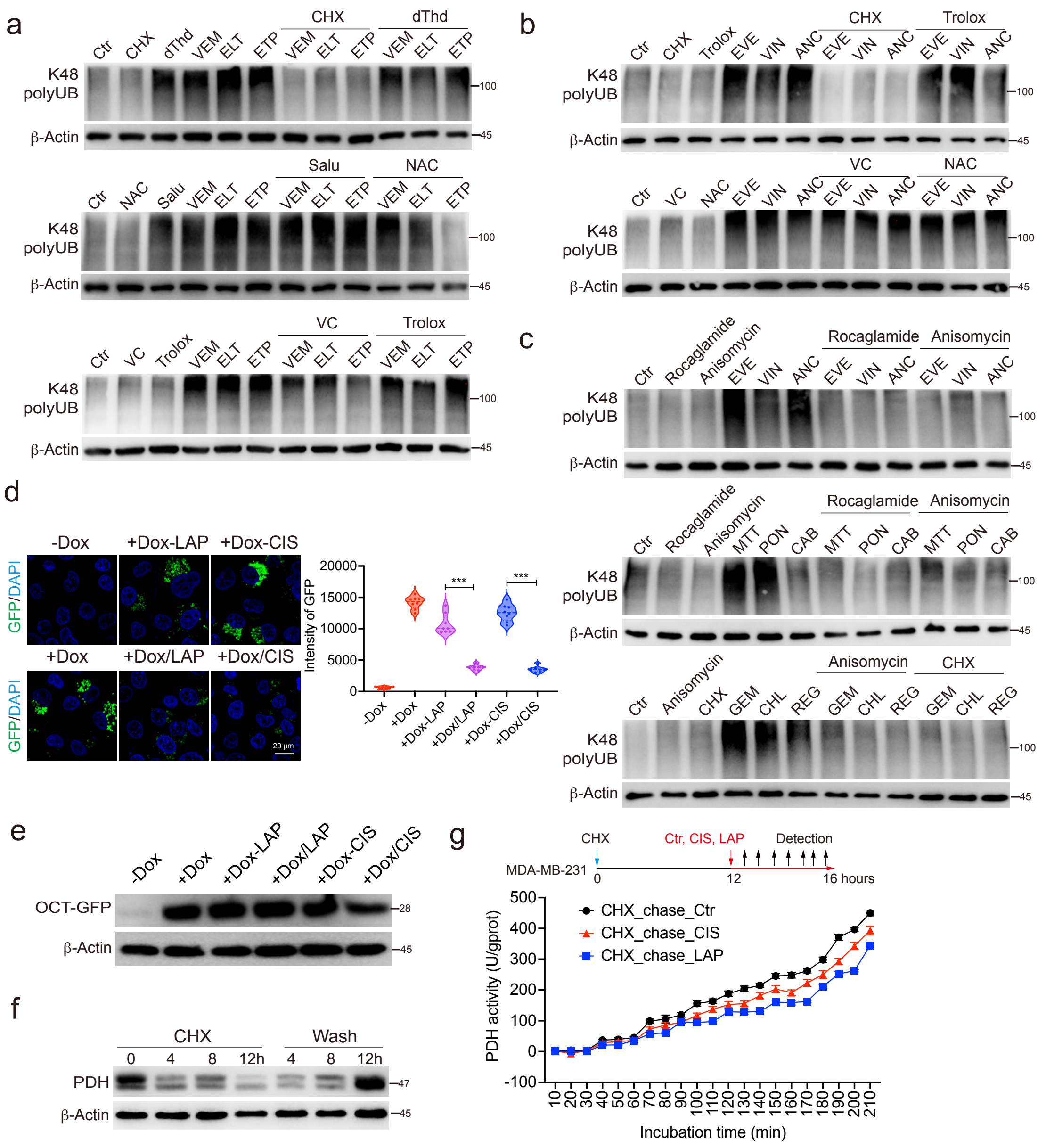

Supplementary Fig S2. Anticancer drugs damage neosynthesized proteins and their functions. (A, B) A549 cells were treated with indicated anticancer drugs or combined with various inhibitors, respectively. K48 polyUB was detected by Western blot. (C) A549 cells were treated with indicated anticancer drugs or combined with Rocaglamide or Anisomycin, and K48 polyUB was detected by Western blot. (D, E) MDA-MB-231-Dox-MTS-GFP cells were treated with indicated drugs, and GFP fluorescence was recorded (D), and expression of GFP was detected by Western blot (E). (F, G) MDA-MB-231 cells were treated with CHX for 24 hours and wash away, expression of PDH and PDH enzymatic activity was chase by Western blot (F) and PDH activity detection kit (G), respectively. All values are presented as mean value (at

least three replications) ± SD, and p value was calculated by comparison with Ctr group or indicated separately (two-tailed Student's t-test, *$p < 0.05$ and **$p < 0.01$).

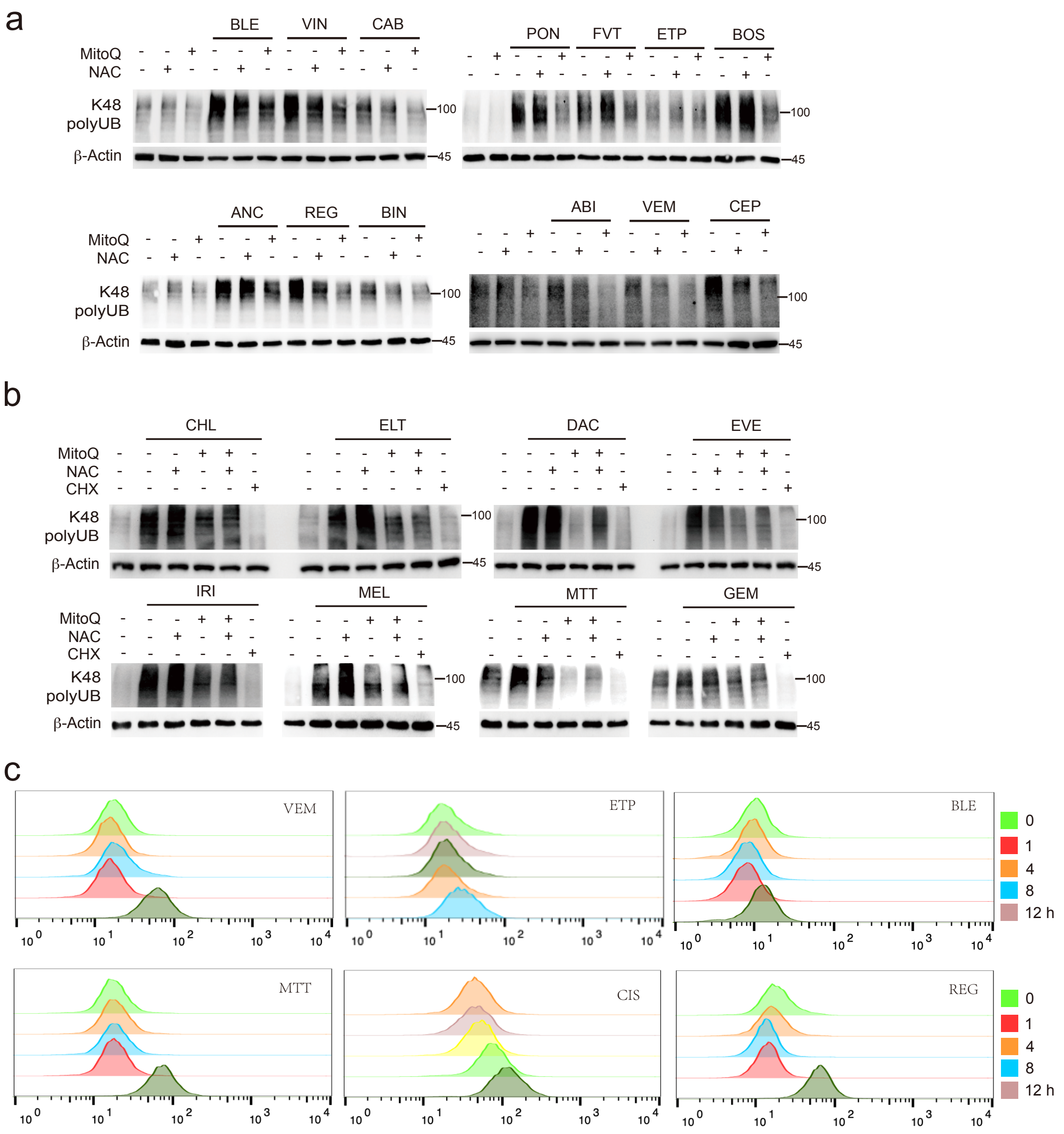

Supplementary Fig S3. Anticancer drugs damage neosynthesized proteins prior to the generation of mtROS that, in turn, enhances protein damage. (A) MDA-MB-231 cells were treated with indicated drugs or combined with NAC or MitoQ, K48 polyUB was detected by western blot. (B) MDA-MB-231 cells were treated with indicated drugs or combined with NAC, MitoQ, or CHX, and K48 polyUB was detected by western blot. (C) MDA-MB-231 cells were treated with indicated drugs for a time course, and cytosol ROS accumulation was detected by DCFDA staining.

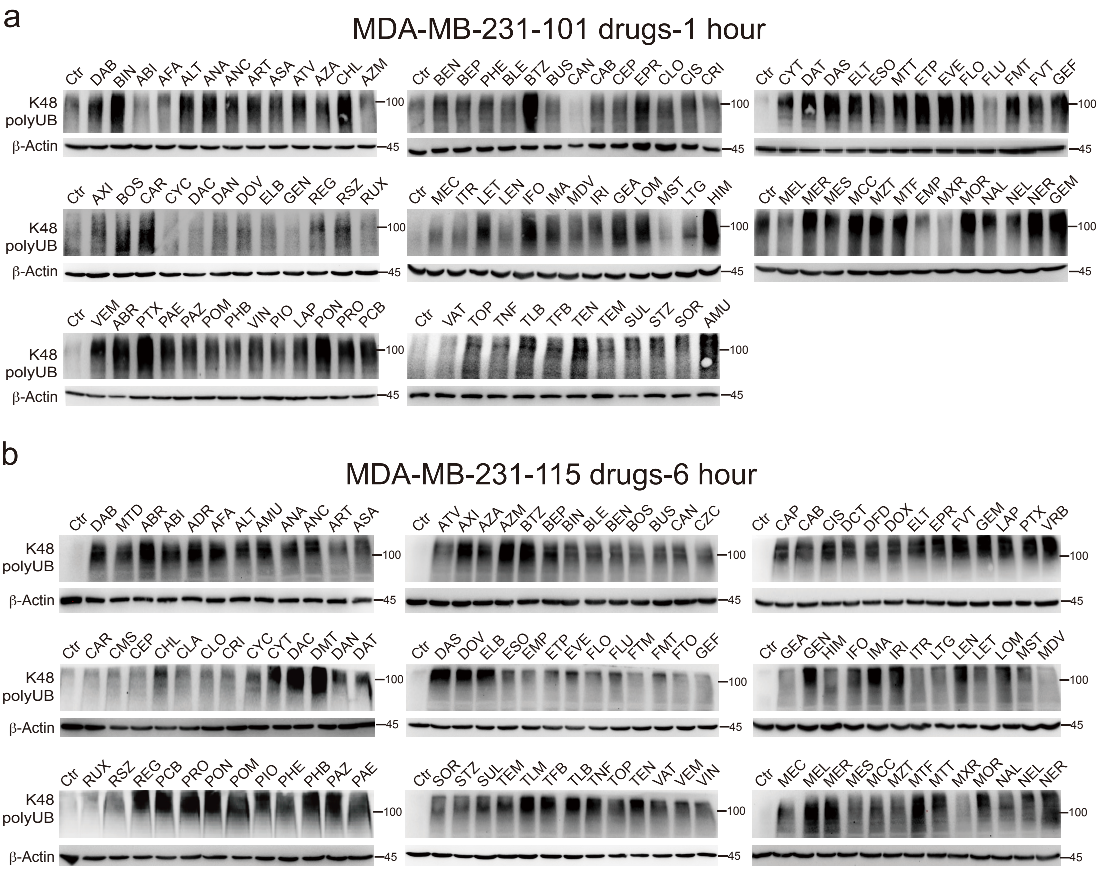
a
MDA-MB-231-101 drugs-1 hour
Ctr DAB BIN ABI AFA ALT ANA ANC ART ASA ATV AZA CHL AZM
Ctr BEN BEP PHE BLE BTZ BUS CAN CAB CEP EPR CLO CIS CRI
Ctr CYT DAT DAS ELT ESO MTT ETP EVE FLO FLU FMT FVT GEF
Ctr AXI BOS CAR CYC DAC DAN DOV ELB GEN REG RSZ RUX
Ctr MEC ITR LET LEN IFO IMA MDV IRI GEA LOM MST LTG HIM
Ctr MEL MER MES MCC MZT MTF EMP MXR MOR NAL NEL NER GEM
Ctr VEM ABR PTX PAE PAZ POM PHB VIN PIO LAP PON PRO PCB
Ctr VAT TOP TNF TLB TFB TEN TEM SUL STZ SOR AMU
K48
polyUB
β-Actin
100
45
b
MDA-MB-231-115 drugs-6 hour
Ctr DAB MTD ABR ABI ADR AFA ALT AMU ANA ANC ART ASA
Ctr ATV AXI AZA AZM BTZ BEP BIN BLE BEN BOS BUS CAN CZC
Ctr CAP CAB CIS DCT DFD DOX ELT EPR FVT GEM LAP PTX VRB
Ctr CAR CMS CEP CHL CLA CLO CRI CYC CYT DAC DMT DAN DAT
Ctr DAS DOV ELB ESO EMP ETP EVE FLO FLU FTM FMT FTO GEF
Ctr GEA GEN HIM IFO IMA IRI ITR LTG LEN LET LOM MST MDV
Ctr RUX RSZ REG PCB PRO PON POM PIO PHE PHB PAZ PAE
Ctr SOR STZ SUL TEM TLM TFB TLB TNF TOP TEN VAT VEM VIN
Ctr MEC MEL MER MES MCC MZT MTF MTT MXR MOR NAL NEL NER
K48
polyUB
β-Actin
100
45

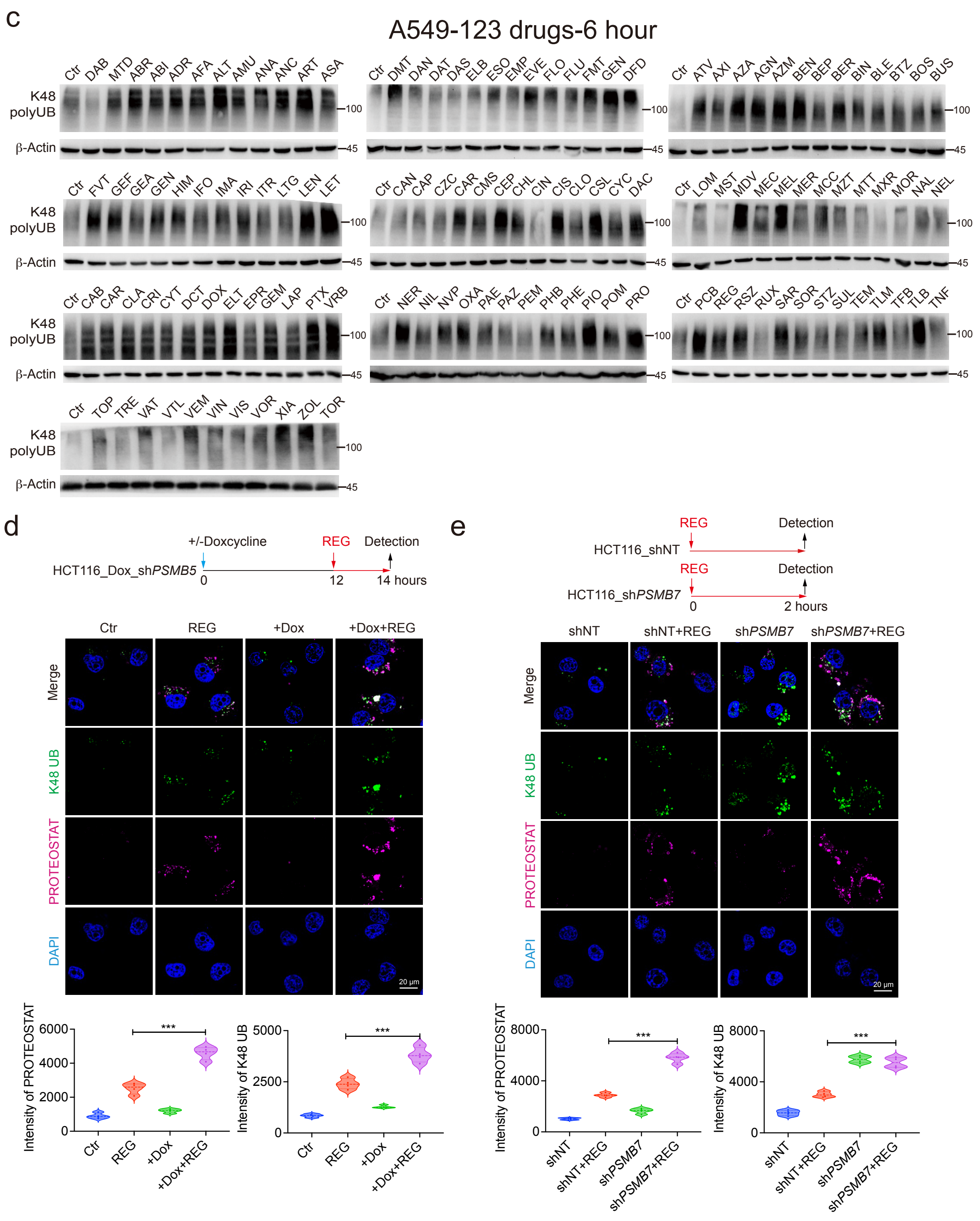

Supplementary Fig S4. Anticancer drug treatment triggers the ubiquitination and proteasome dependent PDR. (A, B) MDA-MB-231 cells were treated with 101 drugs for 1 hour (A) and 115 drugs for 6 hours (B), and K48 polyUB was detected by Western blot. (C) A549 cells were treated with 123 drugs for 6 hours, and K48 polyUB was detected by Western blot. (D, E) HCT116 cells were transfected with shRNA or sgRNA for PSMB7 and PSMB5, respectively, and treat with REG as diagram above, and K48 polyUB and protein aggregation were detected by immunofluorescent staining and the

PROTEOSTAT, respectively. All values are presented as mean value (at least three replications) ± SD, and p value was calculated by comparison with Ctr group or indicated separately (two-tailed Student's t-test, *$p < 0.05$ and **$p < 0.01$).

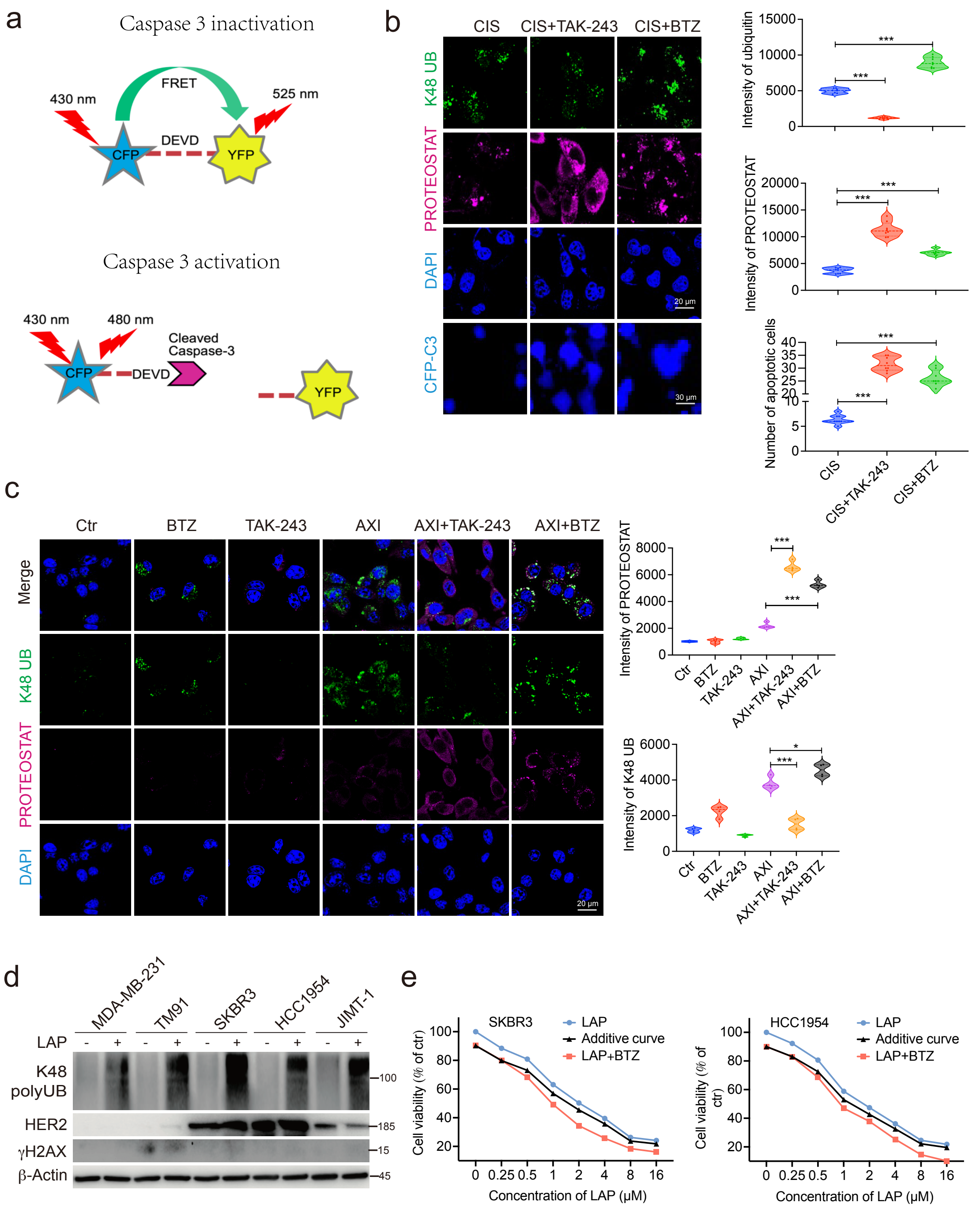

Supplementary Fig S5. Inhibition of the PDR triggers cell death. (A) Principle of caspase-3 reporter Sensor C3. (B) MDA-MB-231 cells were treated with *CIS* or combined with *BTZ* or TAK-243, and K48 polyUB and protein aggregation were detected by immunofluorescent staining and the PROTEOSTAT, respectively. MDA-MB-231-C3 cells were treated with *CIS* or combined with *BTZ* or TAK-243, and caspase 3 activation was monitored by CFP fluorescence. (C) 4T1 cells were treated with *AXI* or combined with *BTZ* or TAK-243, and K48 polyUB and protein aggregation

were detected by immunofluorescent staining and the PROTEOSTAT, respectively. (D) Five breast cancer cell lines were treated with *LAP*, and K48 polyUB, HER2, and γH_2AX were detected by Western blot. (E) Two *LAP* sensitive cell lines were treated with *LAP* alone or combined with *BTZ* treatment, and cell viability was detected by alamar blue assay. Additive curves were calculated by Additive = E1+E2-E1*E2, where E1 is inhibition effect from *LAP* and E2 is inhibition effect from *BTZ* treatment. All values are presented as mean value (at least three replications) ± SD, and p value was calculated by comparison with Ctr group or indicated separately (two-tailed Student's t-test, $*p < 0.05$ and $**p < 0.01$).

a

Patients	ER/PR/HER2/Ki-67 typing	Stages	Treatment before proteasome sampling	Imaging RECIST	Proteasome activity (nmol AMC/min/μL/g)	Treatment after proteasome sampling	Imaging RECIST
1	HER2	IV	Radiotherapy, Capecitabine, Pyrotinib	PD	213.6	Vinorelbine, Herceptin, ADC drug	PD
2	TNBC	IV	Cisplatin, Utidelone	PD	258.3	Capecitabine, Utidelone	PD
3	Luminal	III	Docetaxel, Epirubicin, Cyclophosphamide	SD	32.0	Radiotherapy, Exemestane, Leuprorelin	NA.
4	Luminal	III	Epirubicin, Cyclophosphamide, Docetaxel	PR	3.3	Radiotherapy, Goserelin, Exemestane	NA.
5	TNBC	III	Epirubicin, Cyclophosphamide, Docetaxel	PR	68.7	Radiotherapy, Capecitabine	NA.
6	TNBC	IV	Epirubicin, Cyclophosphamide	PR	82.0	Capecitabine	PD
7	Luminal	III	Epirubicin, Cyclophosphamide, Docetaxel	PR	19.1	Anastrozole	NA.
8	Luminal	IV	Paclitaxel, Carboplatin	PD	150.9	Capecitabine, Halaven, Radiotherapy, Bevacizumab, Paclitaxel	PD
9	Luminal	IV	Docetaxel, Capecitabine	PD	68.1	Doxorubicin, Cyclophosphamide, Eribulin, Apatinib	SD
10	TNBC	III	Epirubicin, Cyclophosphamide, Docetaxel	PR	50.1	Gemcitabine, Sintilimab	PR
11	Luminal	IV	Zoledronic Acid, TDM1	PD	134.4	Trastuzumab, Zoledronic, TDM1, Trastuzumab Emtansine, Fulvestrant	PD
12	TNBC	III	Paclitaxel, Carboplatin	SD	83.4	Gemcitabine	PD
13	TNBC	II	Paclitaxel, Cisplatin	PR	103.4	Capecitabine	PD
14	Luminal	IV	NA.	NA.	32.1	Toripalimab, Paclitaxel	PR
15	Luminal	IV	Cyclophosphamide, Docetaxel, Goserelin, Tamoxifen	PD	265.1	Paclitaxel, Denosumab, Bevacizumab, Trastuzumab	PD
16	TNBC	III	Gemcitabine, Carboplatin, Apatinib	SD	35.3	Apatinib,Capecitabine, Eribulin, Camrelizumab	PD
17	TNBC	IV	Imatinib, Cyclophosphamide, Paclitaxel, Herceptin	PD	188.0	Radiotherapy	NA.
18	HER2	III	NA.	NA.	34.5	Docetaxel, Pertuzumab, Trastuzumab, Capecitabine	PR
19	Luminal	II	NA.	NA.	19.3	Epirubicin, Cyclophosphamide, Paclitaxel	PR
20	TNBC	II	NA.	NA.	52.1	Epirubicin, Cyclophosphamide, Paclitaxel	PR
21	Luminal	II	NA.	NA.	5.2	Pertuzumab, Trastuzumab, Paclitaxel	PR
22	TNBC	IV	Sintilimab, Apatinib, ADC drug	PD	168.4	ADC drug, Capecitabine, Deferoxamine, chemotherapy	PD
23	TNBC	III	Bevacizumab, Sintilimab, Paclitaxel	SD	130.0	Capecitabine, Radiotherapy	PD
24	TNBC	III	Sintilimab, Paclitaxel,	SD	130.6	Capecitabine, Radiotherapy	NA.
25	TNBC	IV	Capecitabine, Exemestane, Herceptin, Pertuzumab	NA.	121.6	Sacituzumabgovitecan, ADC drug	PD

b

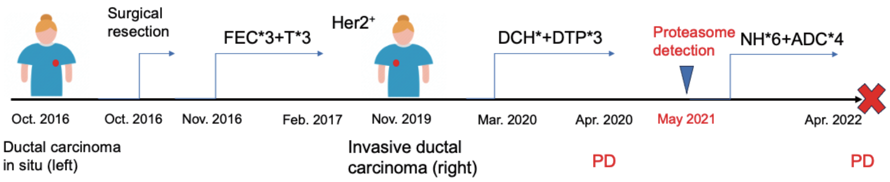

c

Treatment regimen	PD patients	PR/SD patients
FOLFOX	3	3
FOLFOX+Cetuximab	8	6
FOLFOX+Bevacizumab	3	
FOLFIRI	2	1
FOLFIRI+Cetuximab		2
FOLFIRI+Bevacizumab	1	
FOLFOXIRI+Bevacizumab		1
XELOX	5	3
XELOX+Cetuximab	1	
XELOX+Bevacizumab	11	2
Bevacizumab+toripalimab		1
Paclitaxel+Lobaplatin+Raltitrexed		1
Lobaplatin+Raltitrexed	1	
NA.	3	

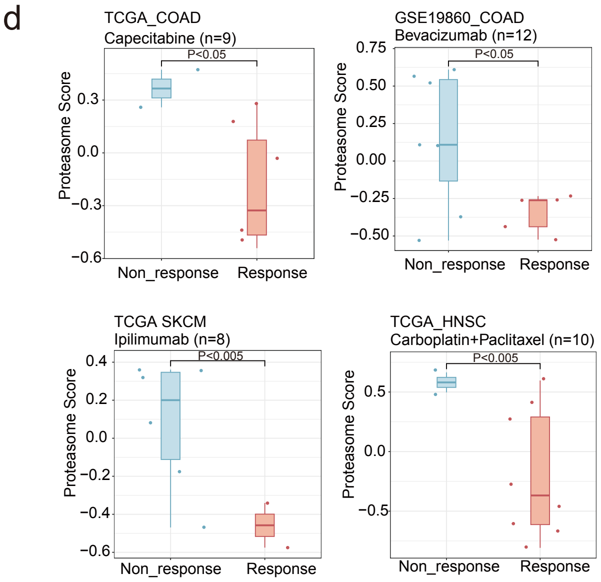

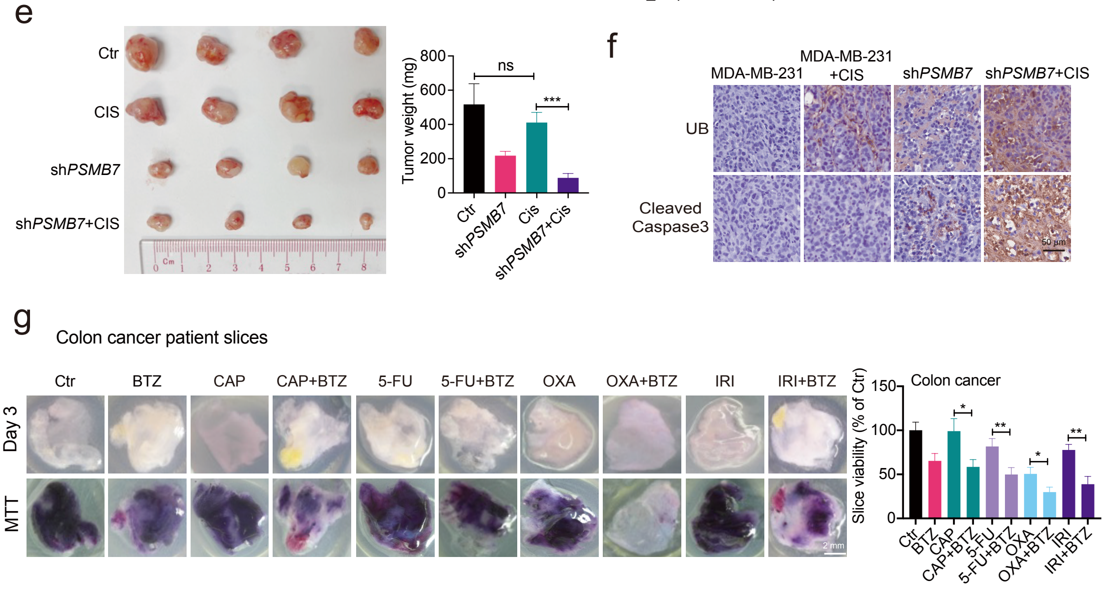

Supplementary Fig S6. Proteasome activity is associated with malignancy and serve as a prediction marker for MDR. (A) The drug treatment regimen for 25 breast cancer patients, the drug response was evaluated by the changes of tumor burden according to the RECIST guideline (version 1.1). (B) Treatment procedure and responses of one breast cancer patient from surgery till proteasome activity detection and the following treatment regime. (C) The drug treatment regimen for 58 colon cancer patients, the drug response was evaluated by the changes of tumor burden according to the RECIST guideline (version 1.1). (D) Patients from TCGA and GES databases, the drug treatment response and the proteasome activity score. (E, F) Nude mice bearing MDA-MB-231 or MDA-MB-231-shPSMB7 cells were treated with vehicle or *CIS* (3

mg/kg) every 3 days for 7 times, and tumor photograph and tumor weight were recorded (E), K48 polyUB and cleaved caspase 3 for tumors were detected by IHC staining (F). (G) One colon cancer patient at stage IV were prepared for tumor slices and treated with the indicated drugs or in combination with BTZ. Slice viability was assessed using MTT staining. All values are presented as mean value (at least three replications) ± SD, and p value was calculated by comparison with Ctr group or indicated separately (two-tailed Student's t-test, $*p < 0.05$ and $**p < 0.01$).

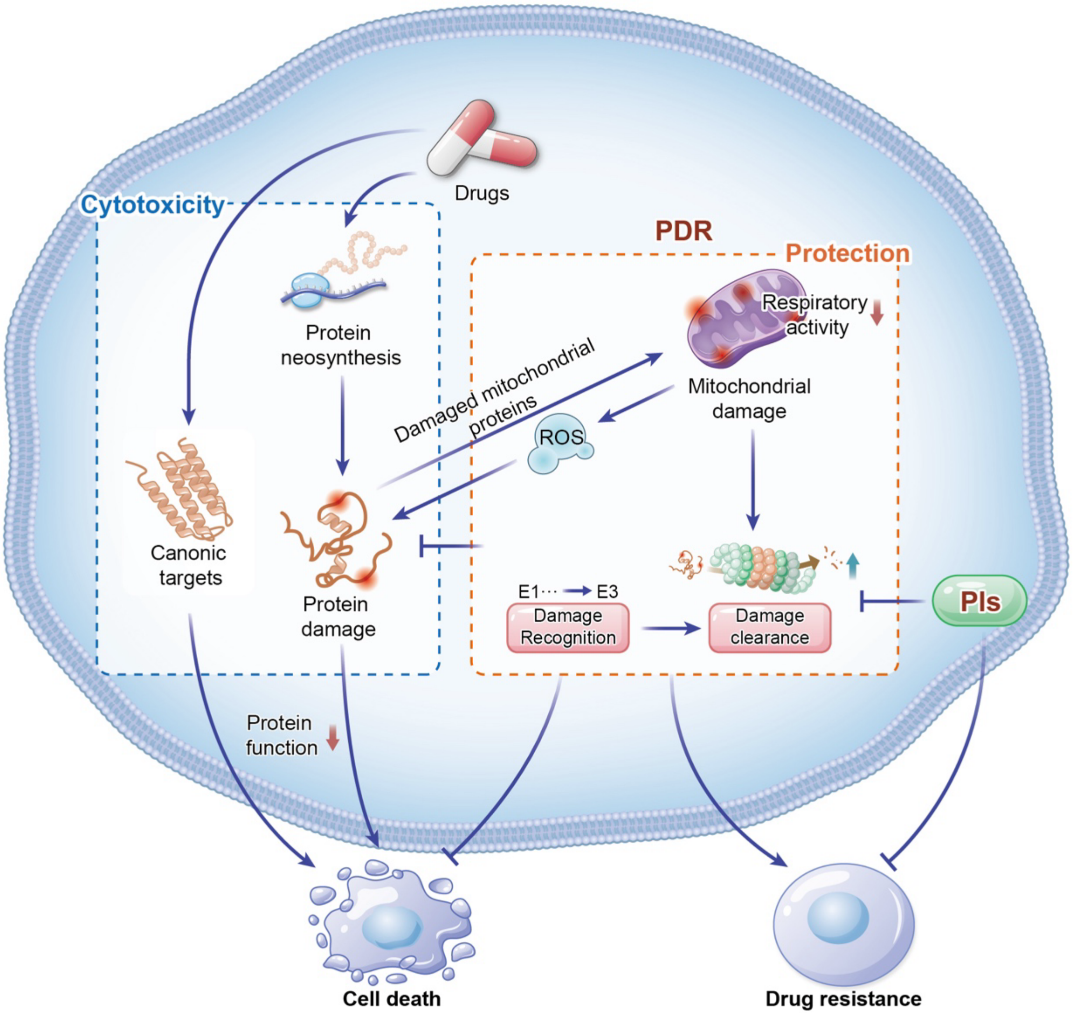

Supplementary Fig S7. graphic abstract

Supplementary Table 1: anticancer drugs used to screen for the protein aggregation and ubiquitination in this study.

Drug Name	Abbreviation	Target/Mechanism	Brand	Cat.#
10-Deacetylbaccatin	DAB	Microtubule	Selleckchem	S2409
Abiraterone	ABR	Androgen/estrogen pathway	Selleckchem	S2246
Abitrexate	ABI	DNA/RNA Synthesis	Selleckchem	S1210
Afatinib	AFA	Tyrosine kinase	Selleckchem	S1011
Altretamine	ALT	DNA binding	Selleckchem	S1278
Amuvatinib	AMU	Tyrosine kinase	Selleckchem	S1244
Anastrozole	ANA	Androgen/estrogen pathway	Selleckchem	S1188
Ancitabine	ANC	DNA/RNA Synthesis	Sigma	A8598
Artemether	ART	ROS	Selleckchem	S2264
Aspirin	ASA	COX enzyme/prostaglandin	Selleckchem	S3017
Atazanavir	ATV	Antiviral	Selleckchem	S1457
Axitinib	AXI	Tyrosine kinase	Selleckchem	S1005
Azacitidine	AZA	DNA/RNA Synthesis	Selleckchem	S1782
Azithromycin	AZM	Bacterial ribosome	Selleckchem	S1835
Bendamustine	BEN	DNA binding	Selleckchem	S1212
Bepotastine	BEP	Anti-inflammatory	Selleckchem	S3037
Bindarit	BIN	DNA binding	Selleckchem	S3032
Bleomycin	BLE	DNA binding	Selleckchem	S1214
Bortezomib	BTZ	Proteasome	BOC Sciences	179324-69
Bosutinib	BOS	Tyrosine kinase	Selleckchem	S1014
Busulfan	BUS	DNA binding	Selleckchem	S1692
Cantharidin	CAN	PP2A	Sigma	C7632
Carboplatin	CAB	DNA binding	Selleckchem	S1156
Carmofur	CAR	DNA/RNA Synthesis	Selleckchem	S1289
Cephalomannine	CEP	Microtubule	Selleckchem	S2408
Chlorambucil	CHL	DNA binding	Selleckchem	S4288
Cisplatin	CIS	DNA binding	Sigma	P4394
Clofarabine	CLO	DNA/RNA Synthesis	Selleckchem	S1218
Crizotinib	CRI	Tyrosine kinase	Selleckchem	S1068
Cyclophosphamide	CYC	DNA binding	Selleckchem	S2057
Cytarabine	CYT	DNA/RNA Synthesis	Selleckchem	S1648
Dacarbazine	DAC	DNA binding	Selleckchem	S1221
Danusertib	DAN	Tyrosine kinase	Selleckchem	S1107
DAPT	DAT	γ-secretase	Selleckchem	S2215
Dasatinib	DAS	Tyrosine kinase	J&K	923898
Dovitinib	DOV	Tyrosine kinase	Selleckchem	S1018
Eltrombopag	ELT	c-Mpl receptor	Selleckchem	S2229
Epirubicin	EPR	Topoisomerase	J&K	194237
Erlotinib	ELB	Tyrosine kinase	Selleckchem	S1023
Esomeprazole	ESO	Proton pump inhibitor	Selleckchem	S2233
Estramustine	EMP	DNA binding	Sigma	E0407
Etoposide	ETP	Topoisomerase	Selleckchem	S1225
Everolimus	EVE	mTOR pathway	Selleckchem	S1120
Floxuridine	FLO	DNA/RNA Synthesis	Selleckchem	S1299
Fludarabine	FLU	DNA/RNA Synthesis	Selleckchem	S1491
Formestane	FMT	Androgen/estrogen pathway	Selleckchem	S1300
Fulvestrant	FVT	Androgen/estrogen pathway	Selleckchem	S1191
Gefitinib	GEF	Tyrosine kinase	Selleckchem	S1025
Gemcitabine	GEM	DNA/RNA Synthesis	Selleckchem	S1149
Geniposidic	GEA	Anti-inflammatory	Selleckchem	S2413
Genistein	GEN	Androgen/estrogen pathway	Selleckchem	S1342
Histamine	HIM	NA.	Selleckchem	S4118
Ifosfamide	IFO	DNA binding	Selleckchem	S1302

Imatinib	IMA	Tyrosine kinase	Selleckchem	S1026
Irinotecan	IRI	Topoisomerase	Selleckchem	S1198
Itraconazole	ITR	Antifungal	Selleckchem	S2476
Lamotrigine	LTG	Ion channel	Selleckchem	S3024
Lapatinib	LAP	Tyrosine kinase	Selleckchem	S1028
Lenalidomide	LEN	NA.	Selleckchem	S1029
Letrozole	LET	Androgen/estrogen pathway	Selleckchem	S1235
Lomustine	LOM	DNA binding	Selleckchem	S1840
Masitinib	MST	Tyrosine kinase	Selleckchem	S1064
Mdv3100	MDV	Androgen/estrogen pathway	Selleckchem	S1250
Mechlorethamine	MEC	DNA binding	Selleckchem	S4252
Melengestrol	MEL	Androgen/estrogen pathway	Selleckchem	E0742
Mercaptopurine	MER	DNA/RNA Synthesis	Selleckchem	S1305
Mesna	MES	NA.	Selleckchem	S1735
Methacycline	MCC	Bacterial ribosome	Selleckchem	S2527
Methazolastone	MZT	NA.	Selleckchem	S1237
Miltefosine	MTF	Anti-parasitic	Selleckchem	S3056
Mitotane	MTT	NA.	Selleckchem	S1732
Mitoxantrone	MXR	Topoisomerase	Selleckchem	S1889
Moroxydine	MOR	Antiviral	Selleckchem	S2486
Naloxone	NAL	NA.	Selleckchem	S3066
Nelarabine	NEL	DNA/RNA Synthesis	Selleckchem	S1213
Neratinib	NER	Tyrosine kinase	Selleckchem	S2150
Paclitaxel	PTX	Microtubule	Selleckchem	S1150
Paeoniflorin	PAE	NA.	Selleckchem	S2410
Pazopanib	PAZ	Tyrosine kinase	Selleckchem	S1035
Phenylbutazone	PHB	COX enzyme/prostaglandin	Selleckchem	S1654
Phenylbutyric	PHE	HDAC	Selleckchem	S4288
Pioglitazone	PIO	PPAR-γ	Selleckchem	S2590
Pomalidomide	POM	NA.	Selleckchem	S1567
Ponatinib	PON	Tyrosine kinase	Selleckchem	S1490
Proadifen	PRO	P450 Enzymes	Sigma	P1061
Procarbazine	PCB	DNA binding	Selleckchem	S1995
Regorafenib	REG	Tyrosine kinase	Selleckchem	S1178
Rosiglitazone	RSZ	PPAR-γ	Selleckchem	S2556
Ruxolitinib	RUX	Tyrosine kinase	Selleckchem	S1378
Sorafenib	SOR	Tyrosine kinase	Selleckchem	S1040
Streptozotocin	STZ	DNA binding	Selleckchem	S1312
Sulindac	SUL	COX enzyme/prostaglandin	Selleckchem	S2007
Temocapril	TEM	NA.	Selleckchem	S2099
Teniposide	TEN	Topoisomerase	J&K	563738
Tofacitinib	TFB	Tyrosine kinase	Selleckchem	S5001
Tolbutamide	TLB	NA.	Selleckchem	S2443
Tolnaftate	TNF	Antifungal	Selleckchem	S2058
Topotecan	TOP	Topoisomerase	Selleckchem	S1231
Vatalanib	VAT	Tyrosine kinase	Selleckchem	S1101
Vemurafenib	VEM	Tyrosine kinase	Selleckchem	S1267
Vincristine	VIN	Microtubule	Selleckchem	S1241
2-Methoxyestradiol	MTD	Androgen/estrogen pathway	Selleckchem	S1233
Adrucil	ADR	DNA/RNA Synthesis	Selleckchem	S1209
Carbazochrome	CZC	NA.	Selleckchem	S3000
Capecitabine	CAP	DNA/RNA Synthesis	Selleckchem	S1215
Docetaxel	DCT	Microtubule	Selleckchem	S1148
Doxifluridine	DFD	DNA/RNA Synthesis	Selleckchem	S2045
Doxorubicin	DOB	Topoisomerase	Selleckchem	S1208
Vinorelbine	VRB	Microtubule	Selleckchem	S4269
Carmustine	CMS	DNA binding	Sigma	C0400

Cladribine	CLA	DNA/RNA Synthesis	Selleckchem	S1199
Dacomitinib	DMT	Tyrosine kinase	Selleckchem	S2727
Flutamide	FTM	Androgen/estrogen pathway	Selleckchem	S1908
Ftorafur	FTO	DNA/RNA Synthesis	Selleckchem	S1300
Temsirolimus	TLM	mTOR pathway	Selleckchem	S1044
Azaguanine-8	AGN	DNA/RNA Synthesis	Selleckchem	S4194
Bergapten	BER	P450 Enzymes	Selleckchem	S4239
Cephalomannine	CEP	Antibiotic	Selleckchem	S2408
Cinacalcet	CIN	calcium-sensing receptor	Selleckchem	S1260
Clorsulon	CSL	Anti-parasitic	Selleckchem	S2613
Neratinib	NER	Tyrosine kinase	Selleckchem	S2150
Nilotinib	NIL	Tyrosine kinase	Selleckchem	S1033
Nilvadipine	NVP	Calcium Channel	Selleckchem	S2721
Oxaliplatin	OXA	DNA binding	Selleckchem	S1224
Pemetrexed	PEM	DNA/RNA Synthesis	Selleckchem	S7785
Saracatinib	SAR	Tyrosine kinase	Selleckchem	S1006
Tretinoin	TRE	NA.	Selleckchem	S1653
Vandetanib	VAT	Tyrosine kinase	Selleckchem	S1046
Vismodegib	VIS	Hedgehog pathway	Selleckchem	S1082
Vorinostat	VOR	HDAC	Selleckchem	S1047
Xl-184	XIA	Tyrosine kinase	Selleckchem	S1119
Zoledronic	ZOL	NA.	Selleckchem	S1314
Toremifene	TOR	Androgen/estrogen pathway	Selleckchem	S1776

Supplementary Table 2: CIS-Bio pulldown assay, Lip-MS for CIS treatment, and Ub-proteome for CIS treatment identified mitochondrial-related genes and proteasome components.

CIS-Bio_VS_Bio (n=287)		CIS-Bio+BTZ_VS_Bio (n=865)		Lip-MS (n=588)		Ub-proteome (n=669)	
Mitochondria	Proteasome	Mitochondria	Proteasome	Mitochondria	Proteasome	Mitochondria	Proteasome
ARL2	PSMA4	CLPB	PSMA2	NUDT19	PSMC5	ECHS1	PSMA1
MRPL45	PSMA3	ECHS1	PSMA4	ACOT7	PSMC4	PDHA1	PSMB7
LYPLA1	PSMB1	PMPCA	PSMD9	ACSL4	PSMB2	PMPCA	PSMC2
ACAT1	PSMB5	HSD17B10	PSMA3	NIPSNAP2	PSMC2	RPL10A	PSMD6
EFHD1	PSMD4	CYCS	PSMA1	CS	PSMD3	NDUFS1	PSMC6
TMEM11	PSME1	PACSIN2	PSMB7	GLUD1	PSME3	PNPT1	PSMD1
STOM		NDUFS1	PSMC2	CYB5R3		SDHB	PSMC5
FKBP10		ARL2	PSMB6	SOD1		FASN	PSMD8
DBT		NDUFAF4	PSMD6	GOT2		CRLS1	PSMD12
UQCRB		LONP1	PSMA7	SOD2		DGUOK	PSMB3
SCCPDH		PRDX6	PSMB4	OAT		PUSL1	PSMB1
CCDC127		GRPEL1	PSMB5	GPI		MDH1	PSMA5
BCL2L13		LYPLA1	PSMD10	LDHB		PI4KA	PSMC1
RDH11		ACAT1	PSMC6	P4HB		NDUFS8	PSMD14
ALDH5A1		MDH1	PSMF1	FH		ATAD1	
FH		ALDH18A1	PSMB2	ACAA1		SAMM50	
NIPSNAP3A		TPI1	PSMD13	DLAT		MFN2	
NUCB2		ALDH2	PSMD8	HSPD1		SLC25A44	
GSTK1		RAB35	PSMC3	PDHB		LDHB	
DTYMK		EFHD1	PSMD12	MTHFD1		ENDOG	
HK2		GPI	PSMB3	COX4I1		GTPBP3	
TXNRD1		UQCRC1	PSMB1	IDE		ISOC2	
ATPAF1		POLG	PSMA6	RPL35A		NDUFS2	
PTGES2		LETM1	PSME3	HK1		MTHFD1	
GLS		NIPSNAP1	PSME2	ACO1		MTHFD2	
PPA2		MTX1	PSMA5	PAICS		TIMM17A	
NDUFS5		LDHB	PSME1	DTYMK		AKR7A2	
IDH1		PRDX4	PSMD14	ALDH1B1		METTL5	
NNT		TMEM11		ATIC		MTCH1	
TSFM		PUS1		PRDX2		OXA1L	
MTCH2		LACTB2		MDH2		ACO1	
FDPS		ACO2		RPIA		ACLY	
ALDH9A1		GOT2		ACADVL		DNA2	
PPWD1		ETFA		NDUFV1		ADCK2	
		MTHFD2		IDH3G		ARMC10	
		HAGH		ACLY		ALAS1	
		AKR7A2		TPI1		MRPL37	
		FKBP10		HSPE1		AADAT	
		PARK7		RPS15A		SHMT1	
		DBT		RPS18		LAMC1	
		UQCRB		GSTO1		MRPL44	
		CARS2		NUCB2		ACOX3	
		CYC1		MRPS22		ABCD3	
		FIS1		MRPS34		NDUFB10	
		ACO1		PRDX4		GLUD1	
		GRHPR		ENDOG		TMEM70	
		GLOD4		DECR1		MRPL39	
		SCCPDH		TST		NUDT19	
		BCS1L		PCK2		QTRT1	
		CCDC127		PITRM1		BCAT2	
		VDAC1		METAP1D		MFN1	
		VDAC3		SND1		MTO1	
		MDH2		GLRX5		FLAD1	

AK2
DHRS7B
TOMM22
BCL2L13
LONP2
RDH11
HSDL1
SHMT1
ALDH5A1
SLC25A24
IDH3A
FH
NDUFB10
GLUD1
ME2
RPIA
PRDX3
NIPSNAP3A
TRMT1
ACOT13
SFXN1
NUCB2
AK4
SLC25A4
SOD1
GSTK1
SPTLC2
POLDIP2
DIABLO
DTYMK
HADH
TIMM23
IDI1
HK2
TXNRD1
ATPAF1
XPNPEP3
PMPCB
DUT
COA7
IDH3G
TIMM44
PPA2
CPOX
GSTO1
PITRM1
NDUFS5
LAP3
ETFB
IDH1
TBRG4
NNT
NUDT5
PSMA6
TRMT10C
SUCLG2
LARS2
GAPDH

FKBP10
FAM136A
RCC1L
SUCLG2
HSD17B10
NUDT9
MRPS26
NLN
MRPL37
PPA2
OXSM
MRPL16
ACOT9
PUS1

MRPS2
PRELID1
PAICS
IARS2
DCAKD
PITRM1
FASTK
P4HB
GAPDH
ME1
CLPP
NCOA4
ABCB8
ALDH9A1
SHMT2
UQCRC2
CCDC51
MPST

TSFM
NDUFS3
PDHB
MTCH2
ATIC
BAX
RMND1
FDPS
SUCLA2
ALDH9A1
TRAP1
SHMT2
PPWD1
PDE12
OAT
